# DMRT1 is required at two steps of ovarian differentiation in rabbits

**DOI:** 10.1126/sciadv.aeh1087

**Published:** 2026-10-21

**Authors:** Emilie Dujardin, Francis Poulat, Ivan Barberá Aura, Aurélie Dewaele, Marjolaine André, Jennifer McKey, Stéphanie Le Gras, Anne Frambourg, Luc Jouneau, Darío G. Lupiáñez, Béatrice Mandon-Pépin, Eric Pailhoux

**Affiliations:** ^1^ BREED, Université Paris-Saclay, UVSQ, INRAE, 78350 Jouy-en-Josas, France.; ^2^ BREED, École Nationale Vétérinaire d’Alfort, 94700 Maisons-Alfort, France.; ^3^ Institute of Human Genetics, CNRS UMR9002, University of Montpellier, 34396 Montpellier, France.; ^4^ Centro Andaluz de Biología del Desarrollo (CABD), Universidad Pablo de Olavide, Junta de Andalucía, Consejo Superior de Investigaciones Científicas, Seville, Spain.; ^5^ Section of Developmental Biology, Department of Pediatrics, University of Colorado Anschutz Medical Campus, Aurora, CO, USA.; ^6^ GenomEast platform, IGBMC, 67404 Illkirch, France.

## Abstract

The transcription factor Doublesex and Mab-3 Related Transcription Factor 1 (DMRT1) is essential for testis differentiation and male fertility, but less is known about its potential involvement in ovarian differentiation. In mice, *Dmrt1*^−/−^ females are fertile, although they show a reduction of *Stra8* expression. Using our *DMRT1* mutant rabbit model, we analyzed ovarian differentiation when *DMRT1* expression is abolished. First, we observed that during early gonadal development, *DMRT1*^−/−^ gonads display a specific transcriptome different from control ovaries and control testes. Second, we assessed the infertility phenotype of mutant females. DMRT1 is highly expressed in germ cells until meiosis initiation, and its disruption leads to a down-regulation of key genes involved in the meiotic process. Chromatin immunoprecipitation sequencing data highlighted that DMRT1 can bind these genes in the rabbit ovary. Our study using the rabbit model shows that in addition to its role in testicular differentiation, DMRT1 has two important functions in ovarian differentiation: an initial involvement in the differentiation of somatic cells and a pivotal role in the differentiation of germ cells. Our data suggested that DMRT1 could be considered in human health as a gene necessary for female fertility.

## INTRODUCTION

Gonadal differentiation is a key step in sexual development. The primary signal (genetic or environmental) that triggers the genetic program for testicular or ovarian differentiation determines the individual’s sex. By extension, the developmental stages during which gonadal fate specification occurs are referred to as the sex determination process. In vertebrates, the main players governing the testicular or ovarian genetic cascades are conserved, but their roles and hierarchical positions in the cascade vary according to the species under consideration. In mammals and many vertebrates, it is now accepted that testis determination takes place in the embryonic progenitor of the supporting cells, where expression of the *SOX9/Sox9* [*SRY* (Sex-determining region of Y chromosome)–related HMG-box gene 9] gene at a well-defined threshold level induces differentiation of the Sertoli cells and thus initiates testis formation (*1*, *2*). How SOX9 expression is induced and the threshold required to trigger Sertoli cell differentiation appear to be species specific. In placental mammals, the testis-determining gene *SRY/Sry* was jointly identified in humans and mice in the early 1990s (*3*–*5*). Although it seemed obvious that SRY must control the expression of the *Sox9* gene (*2*, *6*), the regulatory regions (named Testis-specific enhancer and Enhancer13) targeted by SRY were only recently found in the mouse, in 2008 and 2018, respectively (*7*–*9*).

In females, it is now known that the *Wt1* (Wilms’ Tumor 1) gene is involved in mouse ovarian determination (*10*). Moreover, the RSPO1 (R-spondin)/WNT4 (WNT family member 4)/β-catenin pathway (*11*–*16*) and the transcription factors FOXL2 (Forkhead box L2) and RUNX1 (RUNX family transcription factor 1) (*17*–*21*) are key drivers of the XX supporting cell differentiation into pregranulosa cells. In parallel, after colonizing the XX genital ridge, germ cells must engage in the oogenesis process. The surrounding environment is crucial because secreted factors by somatic cells can affect germ cell differentiation (*22*). After successive mitotic divisions, DAZL (Deleted in Azoospermia Like) expression enables germ cell licensing (*23*, *24*). Then, the *Stra8* (Stimulated by Retinoic Acid 8) gene is crucial for meiotic initiation (*25*). Germ cells progress to prophase I of meiosis before entering meiotic arrest until ovulation (*26*–*28*). During this blockade, somatic cells surround germ cells and form follicles. The formation of the primordial follicle, one oocyte surrounded by one layer of pregranulosa cells, is a key step for female fertility because the finite pool of primordial follicles constitutes the ovarian reserve responsible for the entire reproductive life span (*29*). In rabbits, estrogens are required for the proper proliferation of coelomic cells and germ cells and subsequent follicle formation (*30*). Hence, the rabbit is considered a species with “delayed meiosis” (fig. S1). On the other hand, mice display “immediate meiosis,” as estrogen synthesis does not begin until after birth, when folliculogenesis occurs (fig. S1) (*31*, *32*). This concept of immediate versus delayed meiosis depending on the mammalian species was previously proposed by A. G. Byskov (*33*, *34*).

Although it is now accepted that DMRT1 (Doublesex and Mab-3 Related Transcription Factor 1) plays a role in testicular differentiation (*35*, *36*) in addition to its known involvement in male fertility (*37*–*40*), less is known about its role in the ovary. Although *Dmrt1*^−/−^ mouse ovaries show reduced *Stra8* expression and follicle numbers, females are fertile. Although DMRT1 binding on the *Stra8* promoter was detected, DMRT1 is not required for female fertility in mice (*41*). In contrast, our previous work revealed the involvement of DMRT1 in rabbit female fertility (*35*). In humans, some mutations in the DMRT1 gene have been linked to female infertility (*42*, *43*), as well as male infertility (*44*–*47*). In the present study, we combine RNA sequencing (RNA-seq) on control and mutant gonads at different developmental stages with chromatin immunoprecipitation sequencing (ChIP-seq) on control gonads to decipher the role of DMRT1 in the rabbit ovary. Using our *DMRT1* mutant rabbit line (*35*), we uncovered a role for DMRT1 in the somatic cells during early ovarian differentiation, and we describe how DMRT1 acts in female germ cells to control meiotic entry. We demonstrate once again that the rabbit model is very useful in deciphering the roles of DMRT1 in the gonad. It is evident from the findings of this study that DMRT1 should be taken into consideration in cases of female infertility in humans and mammalian species other than mice.

## RESULTS

### Early ovarian differentiation is impaired in the absence of DMRT1

We previously showed that *DMRT1* expression starts at 12 days postcoïtum (dpc) in a few somatic cells of the developing rabbit genital crest of both sexes (*35*). By 14 dpc, DMRT1 was detected in almost all gonadal somatic cells in XX gonads (Fig. 1A). This is consistent with our previous investigation in XY gonads at 14 and 15 dpc (*35*). At 16 dpc, fewer female somatic cells expressed *DMRT1* (Fig. 1A), whereas it was restricted to Sertoli cells in males, where it persisted throughout life (*35*). In female gonads, *DMRT1* expression in somatic cells progressively disappeared by 18 dpc (*35*) and became undetectable at 22 dpc in these cell types (Fig. 1A). This decrease was accompanied by an equally progressive increase in the expression of the proovarian gene *FOXL2* (*35*). In addition, DMRT1 was faintly detectable in some germ cells by 20 dpc and became restricted to this cell type from 22 dpc in rabbit ovaries (Fig. 1A). In conclusion, DMRT1 expression in ovarian somatic cells appears to peak between 12 and 16 dpc. This observation leads us to two questions: Does DMRT1 play a role at these stages of ovarian development? If so, what is the role of DMRT1 in the progenitor of ovarian somatic cells?

**Fig. 1.**
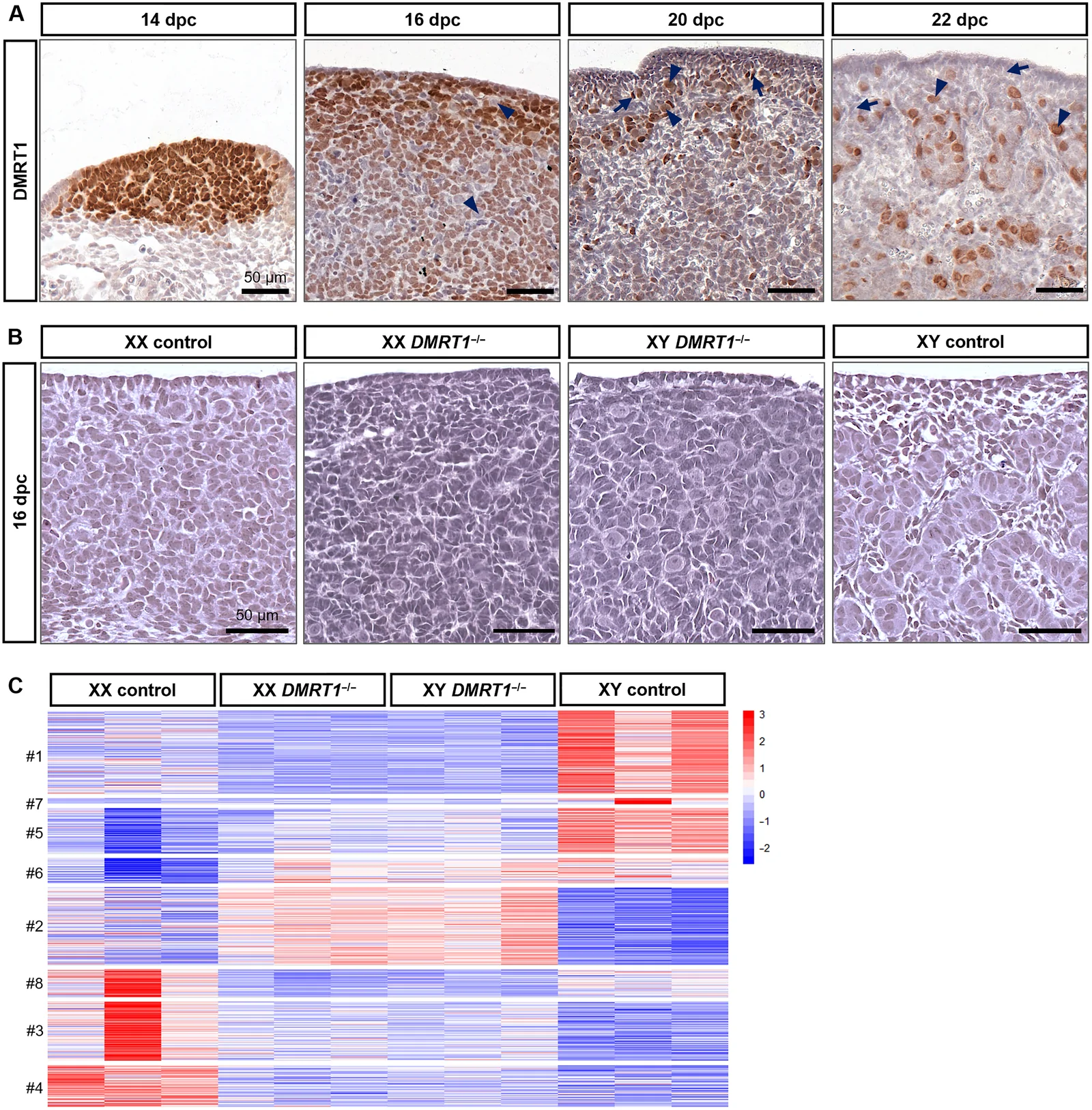
DMRT1 expression in control ovaries and transcriptomic signature of *DMRT1*^−/−^ gonads at 16 dpc. (**A**) DMRT1 immunostaining on XX control ovaries from 14 to 22 (dpc). Arrowhead: germ cells. Arrow: somatic cells. (**B**) Hematoxylin and eosin staining of gonad sections from control and *DMRT1*^−/−^ 16 dpc rabbits. (**C**) Heatmap representation of 921 differentially expressed genes (DEGs; adjusted *P* < 0.05 and |log_2_FC| >1) between XX control, XX *DMRT1*^−/−^, XY *DMRT1*^−/−^, and XY control gonads at 16 dpc.

As previously reported at 20 dpc (*35*), the 16 dpc XY *DMRT1^−/−^* gonads remained morphologically indistinguishable from normal ovaries of the same stage (Fig. 1B). Therefore, to answer the above questions, we first carried out a transcriptomic analysis using bulk RNA-seq of XX and XY control or *DMRT1^−/−^* rabbit gonads at 16 dpc (Fig. 1C). Heatmap representation of the 921 differentially expressed genes (DEGs) in at least one of the four genotypes (adjusted *P* < 0.05 and |log_2_FC| > 1; table S1) was clustered into eight groups (#1 to #8; Fig. 1C and table S1). While only 11.3% of the dysregulated genes at 20 dpc did not correspond to a testicular or ovarian signature (411 of 3640) (*35*), 56% had a profile that was neither testicular nor ovarian (519 of 921). These results show that at 16 dpc, *DMRT1^−/−^* gonads display their own specific transcriptomic signature, irrespective of their chromosomal sex, suggesting that ovarian differentiation is severely disturbed. Thirty-five percent of DEGs (clusters #8, #3, and #4 with 70, 149, and 105 DEGs, respectively) were genes that were highly expressed in control ovaries at 16 dpc but show either delayed up-regulation (*FOXL2*, *LHX2*, or *HSD17B13*, for example) or persistently lower expression (such as *GADD45G*, *HSD11B2*, or *CYP17A1*) in *DMRT1^−/−^* gonads at 20 dpc. Regarding testis-specific genes (clusters #1, #7, #5, and #6, which have 210, 15, 114, and 63 DEGs, respectively, representing 44% of all DEGs), it was noted that most of them (clusters #1, #7, and #5) were not expressed in *DMRT1^−/−^* gonads, and those of cluster #6 were expressed at lower levels compared to XY controls (Fig. 1C). These observations confirm that XY sex reversal occurs as early as the sex determination stage, with no clear evidence of testis differentiation in XY *DMRT1^−/−^* gonads. The 195 remaining genes of cluster #2 (21% of all DEGs) were expressed mainly in *DMRT1^−/−^* gonads (Fig. 1C) and were therefore investigated in more detail. Our initial interpretation was that these genes were genes involved in genital ridge differentiation in both sexes and, therefore, mainly expressed in the supporting cell progenitors, as *DMRT1* expression is strictly somatic before 16 dpc (Fig. 1A).

To confirm our hypothesis, we mapped these 195 genes on a large set of single-cell RNA-seq (scRNA-seq) data obtained on five gonadal developmental stages in rabbits (13, 15, 17, 19, and 21 dpc) of both sexes (*48*). As expected, among 132 genes of the 195 that could be mapped, around two-thirds were expressed by the somatic lineage of the gonads, and some of them, such as *LHX9*, *EMX2*, *NR0B1* (*DAX1*), *KDM3A*, *TOX3*, and *ARX* (figs. S2A and S3), were known for their involvement in genital crest formation and/or early gonadal differentiation (*49*–*54*). Others were well-described ovarian differentiating genes, such as *WNT4*, *RUNX1*, *CYP19A1*, and *ESR1* (figs. S2B and S3) (*16*, *18*, *30*). However, unexpectedly, one-third were expressed by the germinal lineage with *ZBTB16* (*PLZF*), a well-known marker of spermatogonia pluripotency (*55*), and genes involved in the repression of transposable elements such as *MOV10L1*, *TDRD12*, and *TDRD1* (figs. S2C and S3) (*56*–*59*). The expression of the *DMRT3* and *DMRTA1* (*DMRT4*) genes was greatly increased in *DMRT1*^−/−^ gonads. In contrast, these genes were not expressed (*DMRT4*) or very weakly (*DMRT3*) in control gonads of both sexes (table S1). The increased expression of these two genes could be linked to a gene compensation phenomenon.

In summary, we found that ovarian differentiation appears markedly affected at 16 dpc in the absence of DMRT1. Persistent expression of *LHX9* and *EMX2* may indicate a delay in differentiation. This delay was less obvious at 20 dpc (fig. S2A), suggesting that it may only be transient, but we cannot rule out the hypothesis that a developmental delay is at least partially responsible for the ovarian dysfunctions subsequently seen in XX *DMRT1^−/−^* ovaries (*35*).

### DMRT1 and FOXL2 target genes in the somatic cells of rabbit gonads

To better understand the mechanisms underlying the roles of DMRT1 in differentiating somatic cells and compare them with FOXL2, a known key regulator of female somatic cells differentiation (*20*, *21*), we used ChIP-seq of samples from fetal testes and ovaries of 28-dpc control rabbits. This stage corresponds to the end of the period when the ovigerous nests are in place in the female, just before female germ cell meiosis. Three ChIP-seq experiments were carried out in duplicate: DMRT1 on male and female gonads and FOXL2 on female gonads.

FOXL2 ChIP-seq on ovaries produced 12,090 peaks, with a majority (44%) localized in intergenic regions or (43%) within the gene body (4% in exons and 39% in introns). The remaining peaks (12%) were found near the gene, with 10% in promoter–transcription start site (TSS) and 2% in transcription terminal site (TTS; Fig. 2A and table S2). Using the MEME-ChIP tool (*60*), we analyzed the FOXL2 binding motif in the rabbit ovaries. The most significant FOXL2 binding motif (*E* value = 4.0 × 10^−554^) was the same as that described for the mouse (Fig. 2A) (*61*), confirming the specificity of the anti-FOXL2 antibody used and the validity of the results obtained. To determine the functions of FOXL2 target genes, we used the DAVID tool to analyze Gene Ontology (GO) (*62*). Among the 20 main biological processes obtained, we found terms linked to gene expression, embryo development, cell proliferation, and cell-cell signaling by WNT (fig. S4A and table S2). As expected, FOXL2-bound genes were involved in key processes required for gonadal development and seemed closely linked to the WNT-signaling pathway by directly targeting different key genes of this pathway, such as *LEF1*, *AXIN2*, *CTNNB1*, *GSK3B*, *DKK1*, *DKK3*, *LRP6*, *FZD4*, and *WNT5A* (fig. S5).

**Fig. 2.**
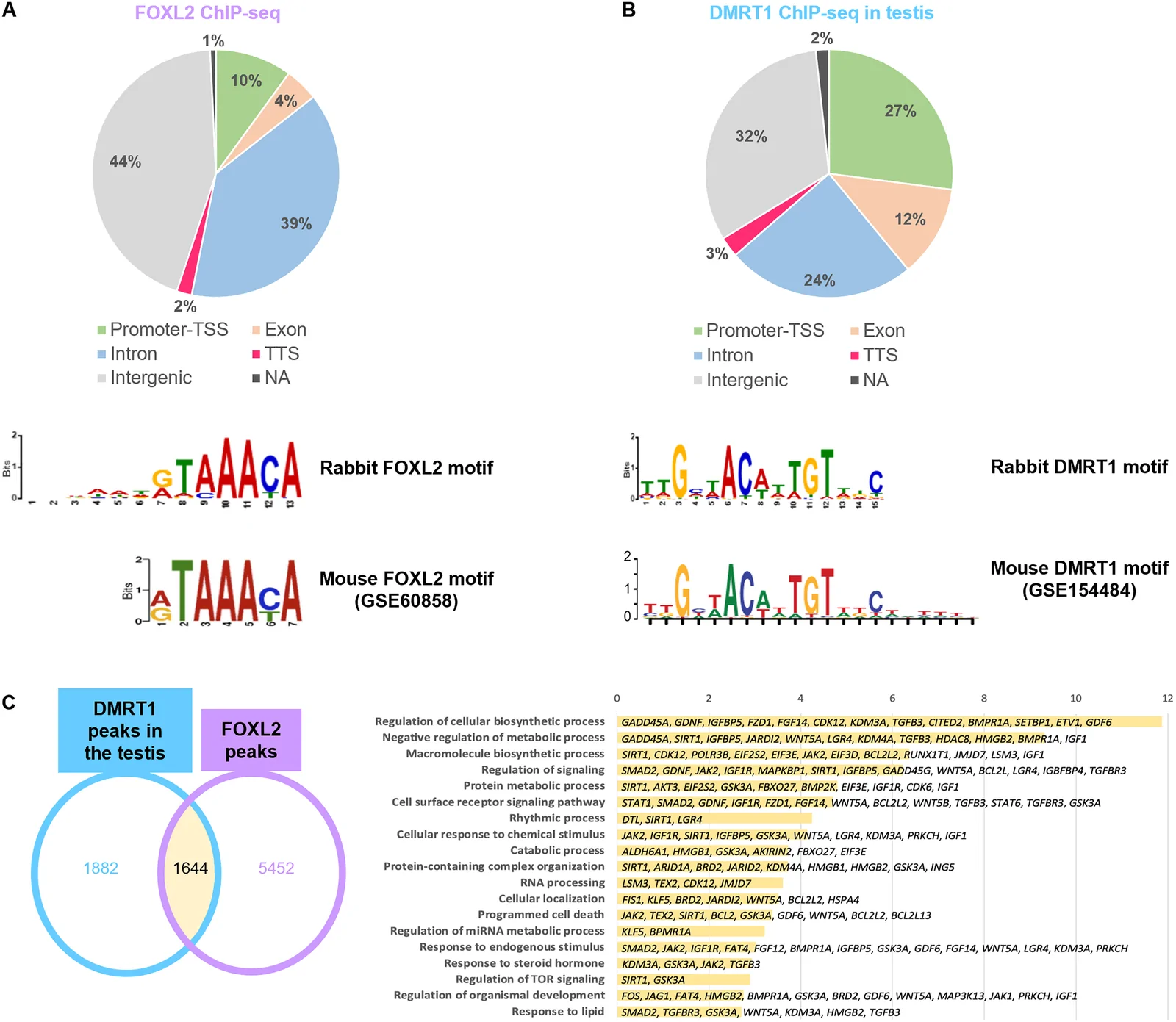
Genome-wide identification of FOXL2-binding and DMRT1-binding regions in rabbit gonads. (**A**) Distribution of FOXL2 peaks on the rabbit genome and the binding motif determined with MEME-ChIP. The top motif (*E* value = 4.0 × 10^−554^) for FOXL2 ChIP-seq in 28-dpc rabbit ovaries is similar to the one described in the mouse (*61*). NA, not annotated. (**B**) Distribution of DMRT1 peaks on the rabbit genome and the binding motif determined with MEME-ChIP. The top motif (*E* value = 5.0 × 10^−244^) for DMRT1 ChIP-seq in 28-dpc rabbit testes corresponds to the one described in the mouse (*63*). (**C**) Venn diagram for genes associated with DMRT1 targets in the rabbit testis and FOXL2 targets in the rabbit ovary, and the top 19 biological processes for common genes determined by DAVID analysis. Terms are classed by their enrichment score, and the most relevant genes for each cluster are shown. miRNA, microRNA.

DMRT1 ChIP-seq on testes produced 4289 peaks, with around one-third (36%) localized within the gene body (12% in exons and 24% in introns), one-third (30%) near the gene (27% in promoter-TSS and 3% in TTS), and one-third (32%) in intergenic regions (Fig. 2B and table S3). The most significant motif (*E* value = 5.0 × 10^−244^) was the same as that described for the mouse (Fig. 2B) (*63*), confirming the specificity of the anti-DMRT1 antibody used and the validity of our results. Using the DAVID tool (*62*) to analyze GO, we found that among the 20 main biological processes, most GO terms were linked to protein modifications, gene expression, chromatin organization, cell death, and regulation of signaling (fig. S4B and table S3). These findings suggest that DMRT1 can modulate key signaling pathways and control apoptosis.

Last, as Enhancer13 was described as the major regulator element of the *Sox9* expression in the testis in mice and because DMRT1 binding sites were identified in the rabbit homologous sequence (*7*), we examined our ChIP-seq data around this region. Although a FOXL2 binding site was observed in the region homologous to mouse Enhancer13 in the rabbit genome, no DMRT1 peak was detected (fig. S6A). We therefore screened the 700 kb upstream of the *SOX9* gene. We found binding of DMRT1 and FOXL2 on the homologous region of the Enhancer33, one of the other SOX9 enhancers previously described in mice (*7*), localized at 2.7 kb upstream of the *SOX9* locus (fig. S6B). Peaks for DMRT1 and FOXL2 were also detected at the *SOX9* promoter (fig. S6B). In addition, a DMRT1 binding site was detected 562 kb upstream of *SOX9*, in the homologous region of RevSex (fig. S6C). This was an interesting finding because in humans, deletion or duplication of this region is associated respectively with 46, XY or 46, XX differences of sexual development with complete gonadal dysgenesis (*64*). In summary, our data show that in the rabbit testis, DMRT1 may regulate the testis-specific expression of *SOX9* by binding the promoter and a 562-kb upstream region, homologous to the RevSex region described in humans. On the other hand, FOXL2 binds at the homologous region of Enhancer13 and at the promoter to inhibit the *SOX9* expression in pregranulosa cells. *Sox9* ovarian transcriptional repression by female-biased factors, such as RUNX1, has recently been demonstrated in the mouse Enhancer13 (*65*).

### Role of DMRT1 in gonadal somatic cells

To try to decipher the role of *DMRT1* in the somatic lineage of the rabbit gonads, we carried out different comparisons of gene lists between ChIP-seq and transcriptomic results. Because *DMRT1* was expressed in Sertoli cells and germ cells in 28-dpc rabbit testes (fig. S7A) and because *FOXL2* expression was restricted to the somatic ovarian lineage (fig. S7B), we compared DMRT1 and FOXL2 gene targets, as FOXL2 is considered to be the antagonist of DMRT1 (*66*). Of 3526 DMRT1 gene targets (without considering the nonannotated peaks), almost half of which (47%) were shared with FOXL2, representing 1644 common gene targets for both factors (Fig. 2C and table S3). For FOXL2, we kept 7096 target genes representing only 58% of FOXL2 peaks. The common gene targets between the two factors represent 23% of the FOXL2 targeted genes, half as many as for DMRT1. Among the 19 main biological processes obtained with the DAVID tool (*62*) on these 1644 common gene targets, we found many terms linked to cell signaling, protein metabolic process, or cell death (Fig. 2C and table S3). Notably, different genes of the transforming growth factor–β (TGF-β) superfamily, known to have implications during gonadal development (*67*, *68*), were in this list (i.e., *TGFB3*, *BMPR1A*, and *SMAD2*), as well as *GDF6*, a factor recently described as the master male-determining gene in several fish species (*69*, *70*).

Last, we compared the dysregulated genes in *DMRT1^−/−^* versus control gonads at 16 dpc (*n* = 1460, considering only the adjusted *P* < 0.05) and at 20 dpc (*n* = 7487, considering only the adjusted *P* < 0.05) (*35*) with DMRT1 target genes (*n* = 2495) or FOXL2 target genes (*n* = 4422) (table S4). We focused on peaks that could be attributed to a gene body (peaks found within the gene or in TSS or TTS, excluding those located in intergenic regions). Among the major genes showing differential expression that were direct targets of FOXL2 or DMRT1, we found well-known players in ovarian differentiation, such as *RSPO1*, *AXIN2*, and *GSK3B* for the β-catenin pathway, as well as *FOXL2* and *CYP26B1*. Four other genes appeared systematically in these comparisons: *GADD45G*, *CITED2*, *HSD11B2*, and *CTNNA2*. The examination of the gene expression dynamics between stages 16 and 20 dpc revealed the presence of three different expression profiles, which were evident in the four gonadal genotypes (fig. S8). The first was represented by *CTNNA2*, a gene completely unknown to us in gonadal differentiation. *CTNNA2*, although weakly expressed, appeared more highly expressed in the testes than in the ovaries, and its expression was lowest in *DMRT1^−/−^* gonads of both sexes (fig. S8). The second was represented by *CITED2*, initially described as a cofactor of WT1 in the regulation of *NR5A1* [Splicing Factor 1 (*SF1*)] in the adrenogonadal primordium (AGP) (*71*). The expression profile of *CITED2* was complex: On the one hand, it seems to be expressed more in the ovaries than in the testes at 16 dpc, but its expression became identical in both sexes by 20 dpc. On the other hand, in *DMRT1^−/−^* gonads, *CITED2* expression was down-regulated at 20 dpc but appeared up-regulated at 16 dpc (fig. S8). *GADD45G* and *HSD11B2* represent the third profile, which represents one of the most notable findings in this study. These two genes were expressed more strongly in ovaries than in testes at both stages studied (16 and 20 dpc), and their expression was down-regulated in *DMRT1^−/−^* gonads of both sexes compared to XX control gonads (fig. S8). *GADD45G* was previously described in mammalian sex determination, with a key role in the male as an activator of *Sry* (*72*, *73*). *HSD11B2* is often described in sex determination in fishes, and its expression was shown to correlate with that of *DMRT1* in a sturgeon species (*74*).

In conclusion, in addition to its key role as an inhibitor of the *FOXL2* gene highlighted in mice (*63*, *75*), DMRT1 appears to be linked to the main signaling pathways already known to be involved in gonadal differentiation, such as the β-catenin pathway or the GADD45G/p38 mitogen-activated protein kinase/GATA4 pathway.

### The number of supporting-like cells is reduced in *DMRT1^−/−^* ovaries

We previously showed that DMRT1 is also expressed at 15 dpc, in a population of PAX8-positive cells located at the border between the mesonephros and the gonad in both sexes (*35*). These cells have the characteristics of supporting-like cells (SLCs) described in mice (*76*). By studying the fate of these SLCs during gonadal differentiation, we observed a reduction in their number at 20 and 22 dpc in *DMRT1^−/−^* ovaries compared to controls (Fig. 3A, fig. S9, and movies S1 to S3). This observation was also attested by the expression levels of PAX8 at 16 and 20 dpc (Fig. 3B). These SLCs, which are at the border between the gonad and mesonephros at 15 dpc (*35*), appeared to invade the female gonad between 15 and 20 dpc. On the other hand, in the testis, they remained localized at the interface between the testis and the mesonephros and at the base of the seminiferous tubules, where they formed the rete testis. Ovary colonization by SLCs appeared to be altered in *DMRT1^−/−^*, with gonadal invasion also varying between *DMRT1^−/−^* XX and XY gonads (Fig. 3A). Curiously, the expression of the *RSPO1* gene exactly matched the degree of invasion of the SLCs: At 16 dpc, *RSPO1* was weakly expressed in the testis and strongly expressed in the ovary (Fig. 3C); in *DMRT1^−/−^* gonads, its expression was intermediate and identical between XX and XY ovaries at 16 dpc (Fig. 3C) but stronger in XX than in XY at 20 dpc (*35*). In addition, the expression of *DKK1*, an antagonist of the β-catenin pathway, showed a profile strictly opposite to that of *RSPO1* in control gonads (Fig. 3C). As it was shown in mice that SLCs express the *WNT4* gene, which is critical for their differentiation (*76*), we confirmed that rabbit SLCs also express *WNT4* (fig. S10). Together, these observations suggest that RSPO1 protein levels are closely associated with the degree of ovarian colonization by SLCs. We thus propose that the quantity of RSPO1 is directly linked to activation of the β-catenin pathway in these SLCs and that activation of this pathway is involved in intragonadal colonization by this cell type.

**Fig. 3.**
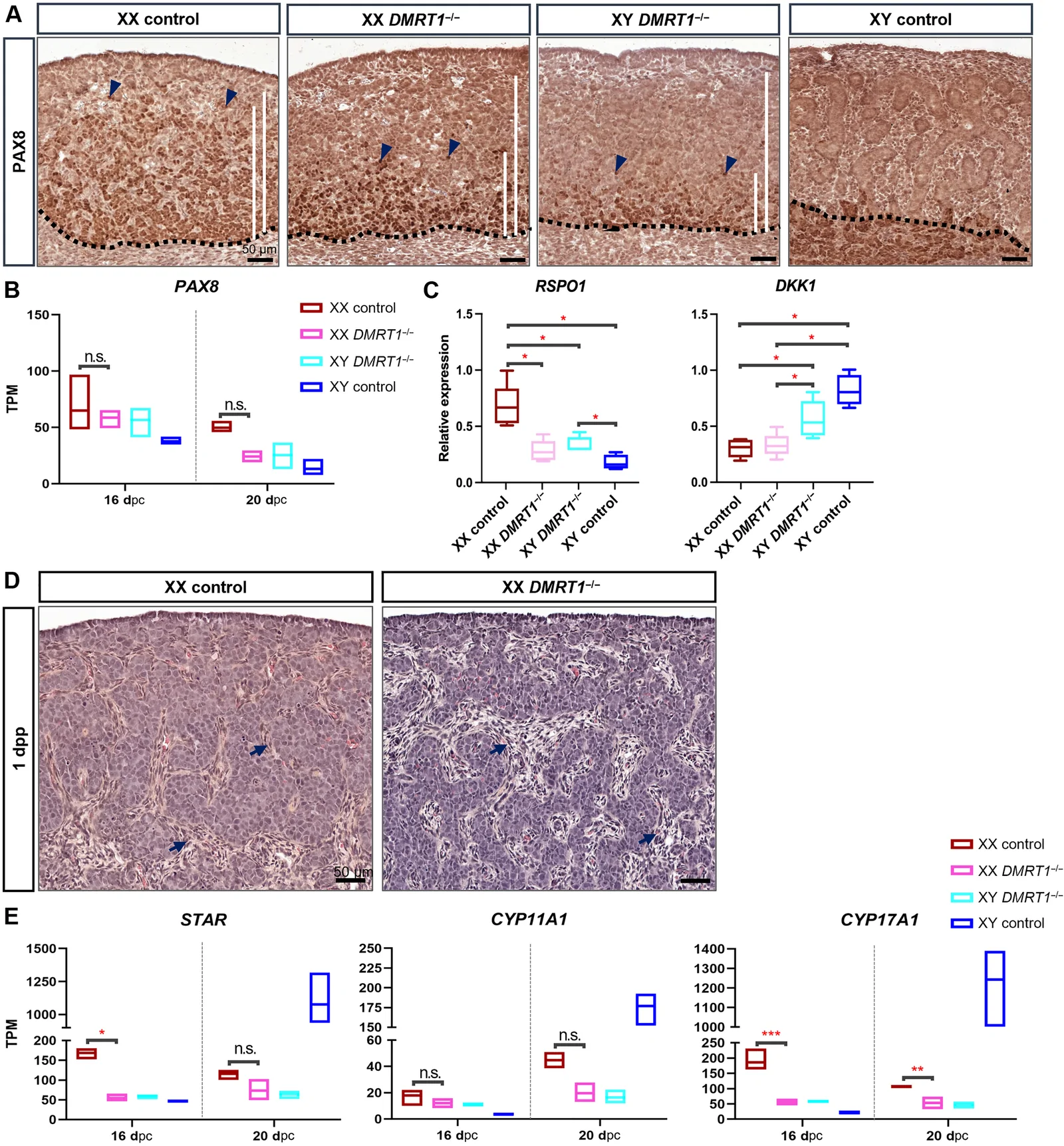
Disrupted colonization of the SLCs and cell proliferation in the absence of *DMRT1*. (**A**) PAX8 immunostaining on XX control, XX *DMRT1*^−/−^, XY *DMRT1*^−/−^, and XY control gonad sections at 20 dpc. Scale bars, 50 μm. Dotted line: delimitation between the gonad and the mesonephros. White lines: extent of the PAX8 expression (left line) versus medullary width (right line). Arrowheads: PAX8^+^ cells. (**B**) Expression data (in TPM) from RNA-seq data (*n* = 3) at 16 and 20 dpc on XX control, XX *DMRT1*^−/−^, XY *DMRT1*^−/−^, and XY control gonads for *PAX8*. Statistical significance was determined using DESeq2 adjusted *P* value: n.s. = not significant. (**C**) Reverse transcription quantitative polymerase chain reaction (RT-qPCR) analyses of *RSPO1* and *DKK1* expression in XX control, XX *DMRT1*^−/−^, XY *DMRT1*^−/−^, and XY control gonads (*n* = 4 to 7) at 16 dpc. Statistical analyses were performed using the nonparametric Kruskal-Wallis test, followed by a pairwise permutation test: *P < 0.05. (**D**) Hematoxylin and eosin staining on gonad sections from XX control and *DMRT1*^−/−^ rabbits at 1 day postpartum (dpp). Scale bars, 50 μm. Arrows: loose fibroblastic tissue. (**E**) Expression data (in TPM) from RNA-seq data (*n* = 3) at 16 and 20 dpc on XX control, XX *DMRT1*^−/−^, XY *DMRT1*^−/−^, and XY control gonads for genes encoding steroidogenesis enzymes (*STAR*, *CYP11A1*, and *CYP17A1*). Statistical significance was determined using DESeq2 adjusted *P* value: *P < 0.05, **P < 0.01, ***P < 0.001, and n.s. = not significant.

### Expression of ovarian steroidogenic enzymes is decreased in *DMRT1^−/−^* ovaries

To better characterize the differentiation between *DMRT1^−/−^* and control ovaries and to link the absence of DMRT1 from the somatic progenitor cells to possible subsequent ovarian phenotypes, we examined the ovaries at birth (Fig. 3D). At this stage, volumetric imaging of intact ovaries showed that *DMRT1^−/−^* ovaries were smaller than those of controls (fig. S11). Histological analysis showed that the ovarian nests are less developed in *DMRT1^−/−^* compared to controls (Fig. 3D and movies S4 and S5). *DMRT1^−/−^* ovaries displayed areas of loose fibroblastic tissue that were absent in controls, as they were filled in by the complete formation of ovigerous nests. This phenotype was very similar, albeit much less marked, to that observed in the absence of estrogens (*30*). This prompted us to evaluate the establishment of early steroidogenesis in the gonads of the different genotypes. We analyzed bulk RNA-seq results obtained at 16 and 20 dpc for all steroidogenic enzymes (Fig. 3E and fig. S12). Three major genes encoding steroidogenic enzymes (*STAR*, *CYP11A1*, and *CYP17A1*) followed the same expression pattern in all genotypes at both stages (Fig. 3E). At 16 dpc, the expression levels of these three enzymes were higher in XX than in XY gonads, when Leydig cell differentiation has not yet begun. The situation was reversed at 20 dpc when the expression levels of these three enzymes were 10 times higher in the testes than in the ovaries of controls. However, the expression of these three enzymes was lower in *DMRT1^−/−^* gonads than in control ovaries at both stages examined. The expression of the other steroidogenic enzymes (*HSD3B1*, *HSD3B2*, and *HSD17B3*) differed from the previous three. They were expressed earlier in the testes (*HSD17B3*) or at the same time (*HSD3B2*) in the gonads of both sexes, and their expression levels remained quite similar between *DMRT1^−/−^* gonads and control ovaries (fig. S12). Together, these results suggest that production of androgens, which can be converted into estrogens by *CYP19A1*, is reduced in *DMRT1^−/−^* ovaries. This leads to lower estrogen production, although *CYP19A1* expression was identical between the two conditions and was even slightly higher in *DMRT1^−/−^* gonads (fig. S2B).

### DMRT1 is crucial for the meiotic engagement of germ cells

We previously showed that germ cells of *DMRT1^−/−^* gonads (XX or XY) are unable to progress into meiotic stages and remain blocked at a premeiotic point (*35*). To define the role of *DMRT1* in XX germ cells, we first established the stage at which DMRT1 is detected in female germ cells. The protein was detectable at around 20 dpc (Fig. 1A) and then remained in most germ cells until birth (Fig. 4A). This 10-day period corresponds to the formation of ovigerous nests under the influence of estrogen secretion from the ovary (*30*). During this period, somatic and germ cells proliferate significantly, resulting in an increased number of DMRT1-positive germ cells (Fig. 4A). Between 1 and 3 days after birth [days postpartum (dpp)], some germ cells stopped expressing *DMRT1*. The number of DMRT1-negative germ cells then increased during the meiotic process until 10 dpp. After meiosis, no more germ cells expressed *DMRT1*, and they are all negative by 18 dpp (Fig. 4A). The first germ cells at the leptotene stage are detectable at birth (1 dpp) (*77*), and 3 days later (3 dpp), some germ cells reach the zygotene stage (fig. S13). On the basis of these data, it seems that the DMRT1 protein was detectable until the leptotene stage and disappeared at the zygotene stage. Ten days after birth, about one-third of all germ cells are in leptotene, one-third in zygotene, and one-third in pachytene (*77*). Our data suggest that DMRT1 is abolished between the leptotene and zygotene stages because approximately one-third of the germ cells remained positive for DMRT1 at 10 dpp (Fig. 4A).

**Fig. 4.**
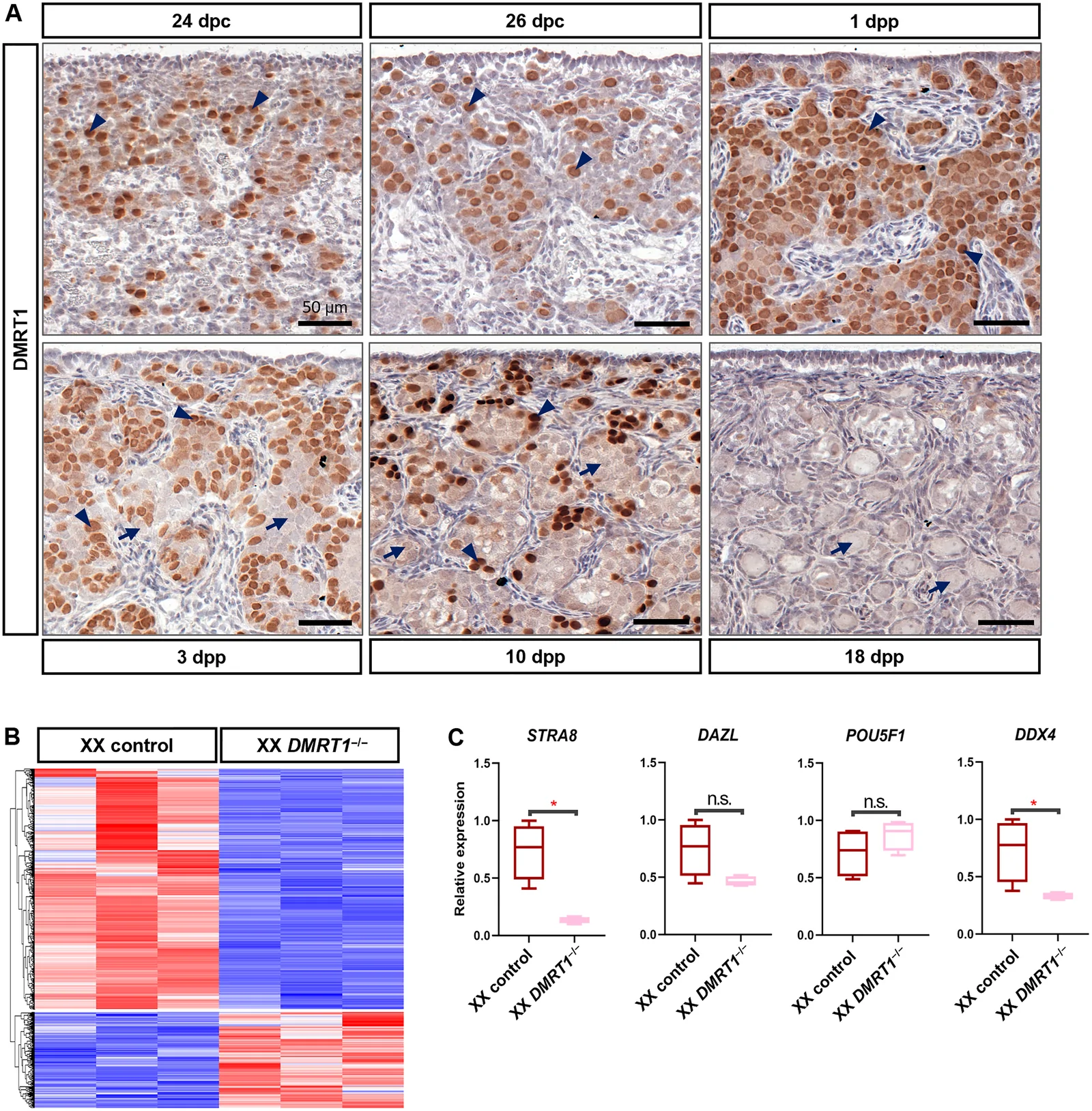
DMRT1 expression in female germ cells is crucial for meiosis. (**A**) DMRT1 immunostaining on XX control gonad sections between 24 dpc and 18 dpp. Scale bars, 50 μm. Arrowheads: germ cells that were expressing DMRT1. Arrows: germ cells that stopped expressing DMRT1. (**B**) Heatmap representation of 1316 DEGs (adjusted *P* < 0.05 and |log_2_FC| >1) between XX control and XX *DMRT1*^−/−^ gonads at 1 dpp. (**C**) RT-qPCR analyses of *STRA8*, *DAZL*, *POU5F1*, and *DDX4* expression in XX control and XX *DMRT1*^−/−^ gonads (*n* = 4) at 1 dpp. Statistical analyses were performed using the nonparametric Mann-Whitney test: *P < 0.05 and n.s.: not significant.

To characterize molecularly the developmental stage at which germ cells become blocked in *DMRT1^−/−^* animals, we performed a transcriptomic analysis of XX *DMRT1^−/−^* gonads versus control ovaries at birth (1 dpp). Heatmap representation of 1316 DEGs (adjusted *P* < 0.05 and |log_2_FC| > 1; table S5) was clustered into two main groups with 942 genes down-regulated in the mutant gonads compared to control ovaries and 374 up-regulated (Fig. 4B and table S5). As expected, among the 942 genes down-regulated in XX *DMRT1^−/−^* gonads, the first three biological processes obtained with the DAVID tool (*62*) (organelle organization, reproductive process, and cell cycle process) contained key genes involved in meiosis, such as *STRA8*, *REC8*, *DMC1*, *SPO11*, *BOLL*, *MEIKIN*, *MAEL*, *SYCP1*, *SYCP2*, and *SYCP3* (fig. S14A). More unexpectedly, among the 374 genes up-regulated in XX *DMRT1^−/−^* gonads were *NANOS2* and *NODAL* (fig. S14B), which are markers for the commitment of germ cells to a male fate (*78*, *79*). These 374 genes overexpressed in mutant gonads also include *POU5F1*, a marker of primordial germ cell pluripotency and survival (*80*). Together, these observations led us to evaluate the expression level of four main markers of the germ cell state, namely, *STRA8*, *DAZL*, *POU5F1*, and *DDX4*, at 1 dpp (Fig. 4C) by reverse transcription quantitative polymerase chain reaction (RT-qPCR). This confirmed that *STRA8* expression was significantly down-regulated in XX *DMRT1^−/−^* gonads. *DAZL* expression appeared to be decreased, and *POU5F1* increased, but not significantly, in the mutant gonads compared to XX ovaries. Last, *DDX4* expression was significantly decreased in XX *DMRT1^−/−^* gonads (Fig. 4C), which suggests either a reduced number of germ cells, a delay in their differentiation process, or both, in the mutant gonads compared to normal ovaries.

In conclusion, transcriptomic analyses performed at 1 dpp confirm that germ cells lacking DMRT1 do not enter meiosis. In addition, the fate of these germ cells appears to be significantly disrupted, with some remaining pluripotent as primordial germ cells and others switching to a male fate.

### DMRT1 targets key meiotic genes in rabbit germ cells

To better understand the role of DMRT1 in germ cell meiosis, we performed ChIP sequencing using rabbit ovaries at 28 dpc (3 days before birth), a stage at which *DMRT1* expression has been restricted to germ cells for 1 week (fig. S5B). We obtained 5288 peaks (i.e., 999 more than in the control testis at the same stage) with a genomic distribution very similar to that obtained in males. One-third (35%) localized within the gene body (11% in exons and 24% in introns), one-third (30%) near the gene (27% in promoter TSS and 3% in TTS), and one-third (32%) in intergenic regions (Fig. 5A and table S6). The DMRT1 binding motif determined by MEME-ChIP (*60*) was very similar to that obtained in testis and previously described for the mouse (Fig. 5A) (*63*). Unexpectedly, given the restricted expression profile of *DMRT1* in germ cells in the ovary compared to the testis at the same stage (fig. S5), of the 20 biological processes found by GO with DAVID (*62*), half were common to both sexes (fig. S15). Most terms encompassed intracellular organization (cytoskeleton organization, regulation of cellular components, and organelle organization), protein modification and protein-DNA complex organization, metabolic and catabolic processes, gene expression, and developmental processes involved in reproduction (fig. S15). Looking closely at the list of genes associated with these biological processes, three observations stood out: (i) *SOX9* was included (as well as *TRIM28*); (ii) numerous genes were linked to the epigenetic machinery (i.e., *SIRT1*, *EZH2*, *SETDB1*, *SETDB2*, *SETD1B*, *METTL3*, *KDM1A*, *KDM3A*, *KDM2B*, *KDM5B*, *DNMT3B*, and *TET1*); and (iii) two key genes were associated with meiosis (*REC8* and *DMC1*).

**Fig. 5.**
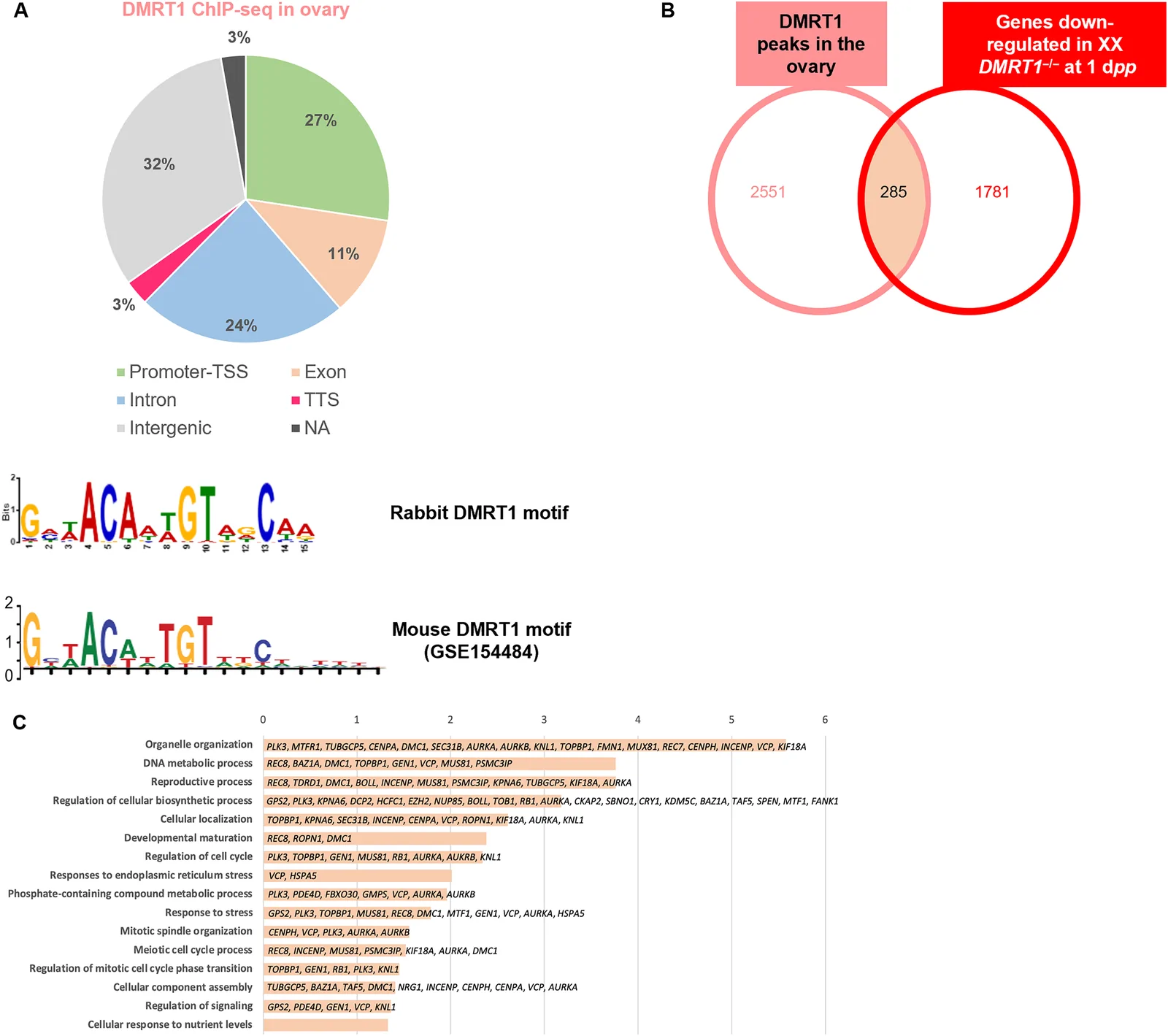
Genome-wide identification of DMRT1-binding regions in the rabbit ovary. (**A**) Distribution of DMRT1 peaks on the rabbit genome and the binding motif determined with MEME-ChIP. The top motif (*E* value = 7.2 × 10^−252^) for DMRT1 ChIP-seq in 28-dpc rabbit ovaries is close to the one described in the mouse. (**B**) Venn diagram for genes associated with DMRT1 targets in the rabbit ovary and genes down-regulated in XX *DMRT1*^−/−^ gonads (adjusted *P* < 0.05) at 1 dpp. (**C**) The top 16 biological processes for common genes determined by DAVID analysis. Terms are classed by their enrichment score, and the most relevant genes for each cluster are shown.

Last, we compared the DMRT1 target genes (*n* = 2836 peaks localized in a gene or in TSS and TTS) with the up-regulated genes in control ovaries versus XX *DMRT1^−/−^* gonads at 1 dpp (*n* = 2066 genes with an adjusted *P* < 0.05 and any fold-change consideration). This comparison yielded 285 shared genes (Fig. 5B). The main biological processes identified using the DAVID tool (*62*) were related to the meiotic process (Fig. 5C). Among the top 16 terms, we found cell cycle regulation, meiotic cell cycle process, DNA metabolism process, and mitotic spindle organization. Among the genes associated with these biological processes, two key meiotic genes, *REC8* and *DMC1*, were included in the list (Fig. 5C). A closer analysis of these two genes showed that their expression was strongly down-regulated in XX *DMRT1^−/−^* gonads (Fig. 6A) and that DMRT1 binding peaks were found at their promoters (Fig. 6B). In addition to *DMC1* and *REC8*, the list included other key genes already known to be involved in meiosis (fig. S16). Examples include *BOLL*, encoding an RNA helicase of the DAZ gene family (*81*); *RB1*, encoding an interacting STRA8 protein (*82*); *CENPH*, involved in meiotic G_2_-M transition (*83*); *CENPA*, encoding a centromere protein critical for chromosome segregation during meiosis (*84*); *AURKA*, encoding a protein kinase involved in spindle building and chromosome segregation (*85*); and *INCENP* and *AURKB*, which promote meiotic sister chromatid cohesion during meiosis (*86*).

**Fig. 6.**
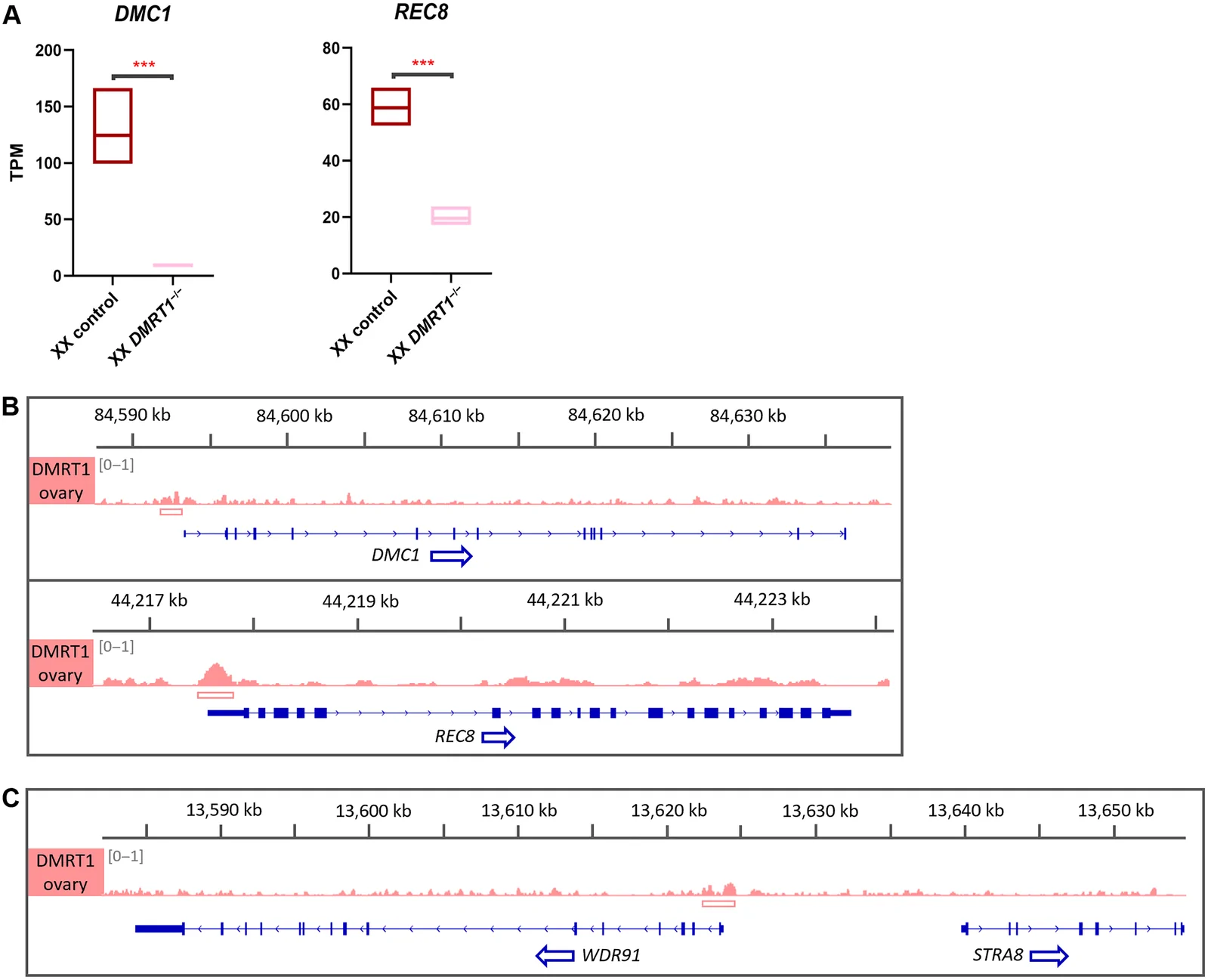
DMRT1 targets key genes involved in meiosis in the rabbit ovary. (**A**) *DMC1* and *REC8* expression data (in TPM) from RNA-seq data (*n* = 3) at 1 dpp on XX control and XX *DMRT1*^−/−^ gonads. Statistical significance was determined using DESeq2 adjusted *P* value: ***P < 0.001. Views of the IGV genome browser for (**B**) *DMC1* and *REC8* or (**C**) *WDR91* and *STRA8* genes bound by DMRT1 in the rabbit ovary (salmon pink). The input background was subtracted with IGV. The rectangles underline relevant peaks.

Last, given that *STRA8* was strongly down-regulated in XX *DMRT1^−/−^* gonads (Fig. 4C) and was previously shown to be a DMRT1 target gene in mice (*41*, *87*), we wondered why it was not detected as a DMRT1 target in our study. To answer this question, we looked at the *STRA8* gene locus in rabbits (Fig. 6C). Although there was no DMRT1 binding peak at the *STRA8* locus, we identified a peak 15 kb upstream of the *STRA8* promoter (Fig. 6C), which could be a distal enhancer for *STRA8*. However, our data do not allow us to determine whether this region regulates *STRA8* expression.

In conclusion, given the number and nature of DMRT1 target genes in rabbit ovarian germ cells at 28 dpc, it is not unexpected that these cells do not enter meiosis. DMRT1 appears to be involved in several premeiotic processes, such as epigenetic remodeling, cell cycle, microtubule network organization, and chromosome segregation. These results raise the question of the stage of development at which these cells come to a halt and whether they begin to duplicate their DNA at the preleptotene stage.

## DISCUSSION

Our study pinpoints critical roles of *DMRT1* in rabbit ovarian development that had not yet been described in other species. In a previous study, we highlighted the involvement of *DMRT1* in rabbit testis determination, and we also showed that *DMRT1* is essential for rabbit female fertility (*35*). In this study, we further characterized the infertility phenotype of the *DMRT1*^−/−^ rabbits, and we also showed that DMRT1 plays a critical role during the first stages of ovarian somatic cell development. As the *Dmrt1*^−/−^ mice were shown to be fertile, albeit with fewer primordial follicles (*41*), this study in rabbits is the first description of *DMRT1*’s critical involvement in ovarian development and function. In humans, one case of primary ovarian insufficiency (POI) was linked to a large 7.6-Mb heterozygous deletion at the 9p locus, where *DMRT1* is located (*42*). More recently, three rare deleterious variants in *DMRT1* were found among a cohort of 104 POI cases (*43*). These observations in humans and our study in rabbits highlight *DMRT1* as a new candidate gene for female fertility, in addition to its already known roles in testicular differentiation and function in numerous species (*35*, *37*).

### DMRT1 is necessary for the differentiation of ovarian somatic progenitors

As we previously published, *DMRT1* expression begins in the rabbit genital ridges of both sexes at 12 dpc, when they start to thicken. In XX developing gonads, *DMRT1* expression in somatic cells starts to decrease with the onset of *FOXL2* expression at 18 dpc (*35*). By analyzing the transcriptome of control and *DMRT1*^−/−^ gonads of both sexes at 16 dpc—a stage at which *DMRT1* expression is restricted to the somatic compartment—using bulk RNA-seq, we showed that XX and XY *DMRT1*^−/−^ gonads are vastly different from XX and XY control gonads. Among the DEGs, we identified several that are known to play a key role in early gonadal differentiation, including *LHX9*, *EMX2*, and *NR0B1* (*49*, *52*–*54*). The up-regulated expression of these genes indicates a potential delay of differentiation in the absence of DMRT1 at 16 dpc and therefore points to an involvement of DMRT1 in the differentiation of ovarian progenitor cells. This situation is somewhat reminiscent of the described role of the *WNT*/*CTNNB1* signaling pathway in mice (*88*), where β-catenin is involved in the proliferation of genital ridges in both sexes, before becoming restricted to ovarian differentiation (*88*). Mirroring the β-catenin pathway, *DMRT1* could also be involved in the differentiation of genital ridges in both sexes before becoming restricted to testicular differentiation.

Our ChIP-seq results also revealed that *GADD45G* is a DMRT1 target. *GADD45G* is expressed during gonadal differentiation, and its expression is affected by the absence of DMRT1. Although the role of this gene has been well documented for the male pathway, where it appears to be a key regulator of *SRY/Sry* (*72*, *73*, *89*), its transcriptional expression is four times higher in developing rabbit ovaries than in testes at 16 and 20 dpc. Thus, *GADD45G* also appears to play a role in the differentiation of supporting cell precursors in both sexes. Its higher expression in female gonads appears to be strongly DMRT1 dependent, as it reverts to the male type in *DMRT1^−/−^* gonads of both XY and XX genotypes at 16 dpc. Furthermore, *GADD45G* expression decreases sharply between 16 and 20 dpc, in line with the decrease in *DMRT1* expression in female gonads.

Another interesting DMRT1 target is the *CTNNA2* gene. This gene was previously described in the early stages of gonadal differentiation in mice, with the highest levels of expression found in the supporting Sertoli cells of the testes (*90*). This previous study found that CTNNA2 is one of 129 cell adhesion molecules playing a key role in tissue organogenesis (*90*). Similar to mice, *CTNNA2* is expressed at higher levels in the testis than in the ovary (a fourfold increase at 16 dpc and a twofold increase at 20 dpc) in our rabbit model. *CTNNA2* expression is markedly reduced in *DMRT1^−/−^* gonads of both genotypes, which could indicate subtle ovarian organogenesis perturbations that are not visible with histology.

### DMRT1 plays a critical role in SLC colonization and ovarian steroidogenesis

Regarding defects that can be identified with histological analysis, we observed disruption in the colonization of *DMRT1^−/−^* ovaries by PAX8-positive SLCs at 20 dpc. Our results suggest that the absence of DMRT1 leads to a lower contribution of the SLCs to the female somatic cell pool. We observed that the localization of the PAX8-positive cells within the gonad was dependent on the genotype. In XX *DMRT1*^−/−^ gonads, SLCs had invaded the gonads to a lesser extent than in XX control ovaries. Moreover, in XY *DMRT1*^−/−^ gonads, they were found within the gonads, while in XY control gonads, they remained at the border between the gonad and the mesonephros. Nevertheless, they still displayed less colonization than in XX *DMRT1*^−/−^ gonads and XX control. The *RSPO1* expression pattern at 16 and 20 dpc followed the different degrees of SLCs invasion in the four genotypes. In other words, the greater the expression of *RSPO1*, the more SLCs entered the gonad and the further they colonized from the medulla to the cortex. As *WNT4* expression was described as a crucial regulator of the SLCs lineage (*76*) and because RSPO1 is often a critical activator of the WNT4/β-catenin pathway (*91*), we propose that DMRT1 can act on *RSPO1* expression to enhance SLC contribution to the ovarian somatic cells in rabbits.

Moreover, we also highlight that ovarian steroidogenesis is affected in the absence of DMRT1. Studying the expression of steroidogenic enzymes at 16 and 20 dpc, the developmental stages at which steroidogenesis begins in both gonadal types, revealed interesting insights. First and somewhat unexpectedly, it appears that steroidogenesis begins earlier in the ovary than in the testis. The transcriptional expression of the three key enzymes *STAR*, *CYP11A*, and *CYP17A1* begins as early as 16 dpc in the ovary, compared to 20 dpc in the testis. Furthermore, the expression of these three enzymes was greatly reduced in *DMRT1*^−/−^ gonads compared to the levels observed in the ovaries at the two stages studied. Given that these three genes are not identified as targets of DMRT1, we hypothesize that this decrease in expression is related to the lower proportion of SLCs in *DMRT1*^−/−^ gonads. This is particularly pertinent, given that SLCs were recently shown to be steroidogenic in rabbit ovaries (*48*). Second, neither *HSD3B1* nor *HSD3B2* is expressed in the ovaries, regardless of the *DMRT1* genotype. This calls into question how these female gonads produce androgens. As we previously demonstrated that *CYP19A1* and estrogens are critical for ovarian development in rabbits (*30*), these gonads should produce some androgens, but the mechanism remains to be found. Third and somewhat unexpectedly, our results reveal that *HSD11B2* exhibits an intriguing expression profile in rabbits. This enzyme is (i) expressed more strongly in the ovary than in the testis, with sexual dimorphism increasing between 16 and 20 dpc; (ii) less expressed in *DMRT1*^−/−^ gonads; and (iii) a direct target of DMRT1. To our knowledge, the role of *HSD11B2* in sex determination has only been documented in nonmammalian species, particularly fish (*74*, *92*). In fish, *HSD11B2* has been linked to the production of 11-ketotestosterone and testicular differentiation and is considered a potential target of DMRT1 (*74*, *92*). The role of this enzyme in the early stages of ovarian steroidogenesis in rabbits remains undefined.

Last, the expression profile of the *CITED2* gene, which could be linked to steroidogenesis, is also interesting. *CITED2* was enriched in ovaries compared to testes at 16 dpc, after which point *CITED2* expression becomes equivalent in both sexes by 20 dpc. Furthermore, *CITED2* expression was disrupted in *DMRT1*^−/−^ gonads, depending on the developmental stage, and we have identified it as a direct target of DMRT1. *Cited2* was primarily shown to act as a WT1 cofactor, increasing the level of *Nr5a1/Sf1* in the AGP to determine adrenal development (*71*). However, it has also been associated with female sex determination in mice, via its role on *Nr5a1/Sf1* (*93*), and with female fertility. CITED2 protein levels in cumulus cells were shown to be a biomarker for the quality of human embryos and pregnancy outcomes (*94*). Furthermore, mutations in this gene can cause POI (*95*). The role of *CITED2* during the early stages of rabbit ovarian differentiation remains to be elucidated, but we hypothesize that its deregulation may be linked to steroidogenesis defects through its close cooperation with NR5A1/SF1.

In conclusion, our study shows that DMRT1 can act at different stages during the development of ovarian somatic cells and has a negative impact on steroidogenesis. These somatic disruptions may also indirectly affect germ cell development and transition to meiosis, in addition to the direct role of DMRT1 in the germline.

### Meiotic initiation of female germ cells is dependent on DMRT1

We previously demonstrated that the absence of DMRT1 in gonads (both XX and XY) results in germ cells failing to enter meiosis. This leads to a failure of the breakdown of the nest and the absence of primordial follicles, resulting in female infertility. At puberty, there are no remaining germ cells in the *DMRT1^−/−^* gonads (*35*). This infertility phenotype was somewhat unexpected, given that previous studies had reported the effects of *Dmrt1* deficiency in female mice (*41*). In mice, most *Dmrt1* mutant germ cells in the fetal ovary exhibit significantly reduced *Stra8* expression and improper localization of SYCP3 and γH2AX during meiotic prophase. The absence of DMRT1 in the fetal ovary leads to a significant reduction of the number of primordial follicles in the juvenile ovary, although this quantity is sufficient to sustain fertility (*41*). To better characterize this marked phenotype in female rabbits, we performed a transcriptome analysis using bulk RNA-seq of ovaries at birth, where we compared XX *DMRT1^−/−^* gonads to controls. We also identified genomic targets of DMRT1 in the germline at 28 dpc, when DMRT1 expression is restricted to germ cells. We demonstrated that the absence of DMRT1 during the 10-day premeiotic period (from 20 dpc to birth, which occurs around 31 dpc) resulted in the down-regulation of a significant number of genes (*n* = 942). As expected, most of these genes were linked to the meiotic process. Furthermore, our ChIP-seq analysis demonstrated that DMRT1 is a key premeiotic factor due to its ability to activate key meiotic genes, such as *RB1*, *REC8*, *DMC1*, or *BOLL (**81**,* *82**,* *96**–**99**)*. In mice, meiotic initiation is linked to the action of *Dazl*, *Stra8*, and retinoic acid (*100*). Nevertheless, *Dmrt1* appears to play a limited role in this process as another activator of *Stra8* (*41*, *87*). In the rabbit model, *DAZL* expression does not appear to be affected by the absence of DMRT1. Its expression is slightly reduced in *DMRT1^−/−^* ovaries, but this likely reflects the fewer germ cells in these gonads rather than a direct effect of DMRT1 on *DAZL* expression. In contrast to mice, it was found that *DMRT1* was critical for meiotic initiation in chickens. It was shown that the germ cells of ZW female chickens lacking DMRT1 are unable to enter meiosis (*36*). These observations suggest that the key role of DMRT1 in germ cell meiosis initiation is not conserved in all species, particularly in mice, where it appears to be less critical for ovarian development. However, the question remains: What about humans? This question remains unanswered, but including the *DMRT1* gene in the list of suspect genes in infertile women would certainly be a good idea, as recently demonstrated (*43*). Moreover, a previous study has already stated that DMRT1 is essential for the germ cell commitment in humans (*101*).

### Limitations of this study

This study provides ample evidence pointing to a pivotal role of the *DMRT1* gene in the ovarian differentiation of rabbits. As with the male component and testicular differentiation (*35*, *37*), it is challenging to determine the precise role of DMRT1 in the ovary due to its sequential expression, first in supporting progenitor cells and then in premeiotic germ cells. As conditional invalidation experiments are hardly feasible in rabbits and the entry of germ cells into meiosis is an asynchronous process (*77*), the *DMRT1^−/−^* ovarian transcriptome should be investigated at different stages (at least 16 dpc and birth) and at the level of individual cell types using scRNA-seq. This approach would facilitate a more comprehensive understanding of the molecular role of DMRT1 in different cell types and would make it possible to precisely quantify the reduction in the number of steroidogenic SLCs at the earliest developmental stage, as well as the number of germ cells at birth. scRNA-seq would also yield information about the different states of differentiation of the germ cells that seem vastly heterogeneous in XX *DMRT1*^−/−^ ovaries. Moreover, as DMRT1 has already been suspected to be a pioneer transcription factor (*63*), single-cell ATAC-seq experiments would also be of interest, especially at 16 dpc, to compare genome-wide chromatin accessibility between the four genotypes.

Meanwhile, this study reveals numerous avenues for further research into the role of *DMRT1* in somatic cells at the outset of ovarian differentiation, as well as the interactions of this gene with previously described molecular pathways. Furthermore, *DMRT1* appears to play a decisive role in the onset of meiosis in the germline, and the targets of this factor described here also open numerous avenues for future investigations.

## MATERIALS AND METHODS

### Animals

New Zealand rabbits (NZ1777, Hypharm, Roussay, France) were bred at the SAJ rabbit facility (Jouy-en-Josas, France). All experiments were performed with the approval of the French Ministry MENESR (accreditation numbers APAFIS#685 and #21451) and following the guidelines issued by the local committee for ethics in animal experimentation (COMETHEA, Jouy-en-Josas). All scientists working directly with the animals had an animal experimentation license issued by the French veterinary services. Hormonal superovulation treatments and surgical embryo transfer procedures were performed as previously described (*102*). The generation of the DMRT1 mutant rabbits was previously described (*35*).

### Histological and immunohistological analyses

Immediately after sampling, whole embryos or gonads were immersed in 4% paraformaldehyde (PFA) in phosphate-buffered saline (PBS) or Bouin’s fixative. After 48 to 96 hours of fixation at 4°C, tissues were washed three times with PBS and stored at 4°C in 70% ethanol until paraffin inclusions. Adjacent sections of 5 μm thick were processed using a microtome (Leica RM2245) and organized on Superfrost Plus Slides (J18000AMNZ, Epredia). Before staining or experiments, sections were deparaffinized and rehydrated in successive baths of xylene and ethanol at room temperature. Hematoxylin-eosin-saffron staining was performed by the @Bridge platform (INRAE, Jouy-en-Josas, France) using an automatic Varistain Slide Stainer (Thermo Fisher Scientific).

In situ hybridization (ISH) was performed using the RNAscope ISH methodology (ACD, Bio-Techne SAS, Rennes, France) when no reliable antibody could be used to characterize the target protein. Briefly, 5-μm sections from PFA-fixed tissue were labeled using RNAscope 2.5 HD assay-brown kit (322310, ACD) and 1000-nucleotide-long probes designed and produced by the manufacturer (the probes used are listed in table S7). Brown labeling was observed as a visible signal, and hybridization was positive when at least one dot was observed in a cell.

Immunohistochemistry was performed using the ABC amplification signal kit (PK-6100, Vector Laboratories) and DAB enzymatic reaction (SK-4100, Vector Laboratories). Briefly, the antigenic sites were unmasked with a citrate buffer (pH 6; H-3300, Vector Laboratories), and endogenous peroxidases were blocked with a 3% H_2_O_2_ solution (H1009, Sigma-Aldrich). Sections were then permeabilized with 1× PBS, 1% bovine serum albumin (A7906, Sigma-Aldrich), and 0.2% Saponin (7395, Merck) and incubated overnight at 4°C with primary antibodies (table S8). Following PBS washes, sections were incubated with biotinylated secondary antibodies (table S8). After the ABC kit incubation and DAB revelation, hematoxylin staining was briefly performed to visualize the whole tissue. All stained sections were scanned using a 3DHISTECH panoramic scanner at the @Bridge platform (INRAE, Jouy-en-Josas, France).

### Whole-mount immunofluorescence

Whole-mount immunofluorescence followed by ethyl-cinnamate tissue clearing was performed as previously described for adult mouse ovaries (*103*). Briefly, ovaries from 22-dpc and 4-dpp female rabbits were dissected in PBS (day 0), fixed for 48 to 96 hours at 4°C in 4% PFA/PBS, and gradually dehydrated in 100% methanol for storage at −20°C. When ready for analysis (day 1), samples were progressively rehydrated to PBS 0.2% Triton X-100 (PTx.2) following an isopropanol gradient: 70% isopropanol/PBS; 50% isopropanol/PBS; 30% isopropanol/PBS; 100% PTx.2. Samples were then permeabilized overnight at 37°C in PTx.2 with 2.3% glycine. On day 2, samples were incubated in blocking solution (PTx.2 10% horse serum solution) for 6 hours at 37°C. Tissues were then incubated in primary antibodies (table S8) diluted in PTx.2 with 0.001% heparin (PTwH) with 10% horse serum for 72 hours at 37°C. On day 5, samples were washed three times for 1 hour at room temperature in PTwH, followed by incubation at 37°C for 48 hours with secondary antibodies (table S8) diluted in PTwH with 10% horse serum. On day 7, after washing three times for 1 hour in PTwH, samples were progressively dehydrated into 100% isopropanol (30% isopropanol/PBS; 50% isopropanol/PBS; 70% isopropanol/PBS; 100% isopropanol) and left in 100% isopropanol overnight. On day 8, samples were cleared in ethyl cinnamate (ECi). Samples were left overnight in ECi to allow for sufficient clearing before imaging.

### Whole-mount image acquisition

ECi-cleared ovaries were mounted in a three-dimensional (3D)–printed coverslip holder (model available for download at www.mckeylab.com/3d-prints) as previously described for adult mouse ovaries (*103*). Briefly, a size 1.5 40 × 22 mm coverglass was sealed to the bottom of the coverslip holder using superglue. Samples were placed in the center of the coverslip, and a sealant barrier was created along the edges of the coverslip holder using silicon sealant gel. A second coverslip was dropped on top of the tissue and compressed just enough to hold the tissue down. The chamber was then filled with ECI using the inlet holes on the coverslip holder. Once the chamber was completely full and free of bubbles, the inlet holes were plugged using silicon sealant. Images were acquired using an Andor Dragonfly (Oxford Instruments, Abingdon, UK) with a Leica DMi8 microscope stand (Leica Microsystems, Wetzlar, Germany). Z-stacks were acquired with a 3-mm interval using a 10× dry objective. We used a high-power laser engine equipped with 637- and 561-nm lasers to visualize Alexa Fluor dye 647 (PAX8) and Cy3 (DDX4/FOXL2), respectively. We used high laser powers (>10%) and low exposure times (<100 ms) to optimize for fast acquisition. Images were processed using the Imaris software (Andor Technology, Belfast, UK) to generate figure displays and 3D segmentations (using the surfaces module). Processed images were assembled into figures using Adobe Photoshop, and Adobe Premiere Rush (San Jose, CA, USA) was used to produce videos.

### Total RNA extraction and RT-qPCR

Immediately after sampling, 16-dpc or 1-dpp gonads were snap frozen in liquid nitrogen and stored at −80°C until extraction. Total RNAs were isolated using TRIzol reagent (15596018, Life Technologies), purified with the RNeasy Micro kit (74004, QIAGEN) or Mini Kit (74104, QIAGEN) following the manufacturer’s instructions, and then deoxyribonuclease treated (1023460, QIAGEN). RNAs were quantified with a Qubit Fluorometric Quantification kit (Q32852, Life Technologies).

RT of 30 ng (16 dpc) or 100 ng (1 dpp) RNAs using the Maxima First-Strand cDNA Synthesis Kit (K1641, Thermo Fisher Scientific) was done. qPCR with diluted cDNA was performed in duplicate for all tested genes with the StepOne system (Applied Biosystems) and Fast SYBR Green Master Mix (4385612, Applied Biosystems). *HPRT*, *H2AFX*, *YWHAZ*, and *SF1* were used as the reference genes to normalize the results with qBase^+^ software (Biogazelle NV, Ghent, Belgium). The sequences of the primers used are listed in table S9.

For each experiment, values were plotted using GraphPad Prism Software (GraphPad Software Inc., La Jolla, CA, USA). Statistical data analyses from control and *DMRT1*^−/−^ gonads at 16 dpc and 1 dpp were performed using the R Studio software. Because of the small number of samples in each group, comparisons were made using the Kruskal-Wallis rank sum test, followed by pairwise permutation *t* tests (1000 permutations; *P* value adjusted with the Benjamin-Hochberg method) for the data obtained at 16 dpc or using the Mann-Whitney test for the data obtained at 1 dpp.

### RNA-seq and bioinformatics analysis

Total RNAs were extracted from control and *DMRT1*^−/−^ rabbit gonads at 16 dpc or 1 dpp (*n* = 3 for each phenotype and each sex). Total RNA quality was verified on an Agilent 2100 Bioanalyzer (Matriks, Norway), and samples with an RIN > 9 were made available for RNA sequencing. This work benefited from the facilities and expertise of the I2BC High-throughput Sequencing Platform (www.i2bc.paris-saclay.fr/sequencing/ng-sequencing/; Université Paris-Saclay, Gif-sur-Yvette, France) for oriented library preparation (Illumina TruSeq RNA Sample Preparation Kit) and sequencing (Paired-end 50–35 bp and NextSeq500). More than 35 million 50- to 35–base pair (bp) paired-end reads and more than 23 million 51-bp reads were generated for each 1-dpp and 16-dpc samples, respectively. Demultiplexing and adapter removal were performed at the I2BC High-throughput Sequencing Platform using bcl2fastq2-2.18.12 from 1-dpp gonads or bclconvert 3.9.3 from 16-dpc gonads and Cutadapt 3.2. Only reads longer than 10 pb were used for the subsequent analysis. Quality control of raw RNA-seq data was processed by FastQC v0.11.5.

Reads were mapped on all the genes of a better-annotated rabbit genome, as described in Dujardin *et al*. After mapping with STAR version 2.5.1b (*104*), reads were counted using FeatureCounts version 1.4.5 (*105*). Data normalization and single-gene level analyses of differential expression were performed using DESeq2 (*106*). Differences were considered to be significant for Benjamini-Hochberg adjusted *P* < 0.05 and absolute log2FC > 1 (*107*). RNA-seq data have been deposited via the SRA Submission portal (www.ncbi.nlm.nih.gov/sra/PRJNA1347743), BioProject ID PRJNA1347743.

Ontology analysis was performed with the DAVID tool (*62*) v2021. The Ensembl ID of genes with adjusted *P* < 0.05 and |log2FC > 1| was used for each analysis. Analyses were performed on gene sets from the Gene Ontology Biological Process category, and each term with an enrichment score > 1.3 was retained.

### ChIP sequencing, bioinformatics, and ontology analyses

The stage of 28 dpc was chosen for practical reasons. We were able to obtain the necessary number of gonads (*n* = 50 per sex) from an existing protocol investigating uterine scarring following cesarean sections, in full accordance with the 3Rs principle.

Immediately after sampling, gonads were snap frozen in liquid nitrogen and stored at −80°C. For the ChIP analysis, 50 testes and 50 ovaries were cold ground with a mortar cooled by liquid nitrogen (H37260-0100, Bel-Art). The ChIP protocol is derived from one of the “Low Cell ChIPseq” kits (53084, Active Motif). Powdered tissue was immediately fixed with PBS/1% formaldehyde for 20 min at room temperature. Fixation was stopped with glycine solution included in the kit. After washing, powdered tissue was homogenized with an ULTRA-TURRAX for 30 s at 9000 rpm. Cell lysis was completed with a Dounce homogenizer. Sonication was performed in a Covaris E220evolution with microTUBE-500 AFA (520185). ChIP and sequencing libraries were prepared using the Low Cell ChIP-seq Kit and the Next Gen DNA Library Kit (Active Motif), which contains molecular identifiers used to remove PCR duplicates from sequencing data. For each condition, 20 μg of sheared chromatin was precipitated in duplicates with DMRT1 (*108*) or FOXL2 antibodies (F.P.). Reads were mapped to the same *Oryctolagus cuniculus* genome that had been used for the RNA-seq analyses using Bowtie2 (*109*) v2.4.4 with default parameters except for “-X 2000.” The tool rmDupByMids.pl provided by Active Motif was used to remove duplicated reads. BAM files were sorted, and reads with a mapping quality below 10 were filtered out using SAMtools view and sort (*110*) v1.15.1. BigWig files were generated using deepTools bamCoverage (*111*) v3.5 with the following parameters: -bs 10 --normalizeUsing CPM --effectiveGenomeSize 2395757213 --skipNonCoveredRegions --extendReads. Peak calling was performed with spp v1.15.5 using the ENCODE ChIP-seq v2.0.1 pipeline (https://github.com/ENCODE-DCC/chip-seq-pipeline2). Peak set retained for further analyses is the IDR/optimal ones. Peaks were annotated using Homer annotatePeaks.pl (*112*) v4.11.1 with Ensembl annotations derived from release 106. Functional enrichment analysis was performed using DAVID (*62*) v2021. The Ensembl ID of genes associated with peaks in introns, exons, promoter-TSS, and TTS was used for each analysis. Analyses were performed on gene sets from the Gene Ontology Biological Process category, and each term with an enrichment score > 1.3 was retained.
