# Supplementary material for: DMRT1 is required at two steps of ovarian differentiation in rabbits
Source: Sci Adv. 2026 Oct 21;12(43):eaeh1087. doi: 10.1126/sciadv.aeh1087 (PMC13685955; doi:10.1126/sciadv.aeh1087)
Supplement: Supplementary file 1 [file sciadv.aeh1087_sm.pdf]

Science Advances
AAAS

Supplementary Materials for

DMRT1 is required at two steps of ovarian differentiation in rabbits

Emilie Dujardin *et al.*

Corresponding author: Emilie Dujardin, edujardin@kumc.edu; Eric Pailhoux, eric.pailhoux@inrae.fr

Sci. Adv. **12**, eaeh1087 (2026)
DOI: 10.1126/sciadv.aeh1087

The PDF file includes:

Figs. S1 to S16
Legends for tables S1 to S6
Tables S7 to S9
Legends for movies S1 to S5

Other Supplementary Material for this manuscript includes the following:

Tables S1 to S6
Movies S1 to S5

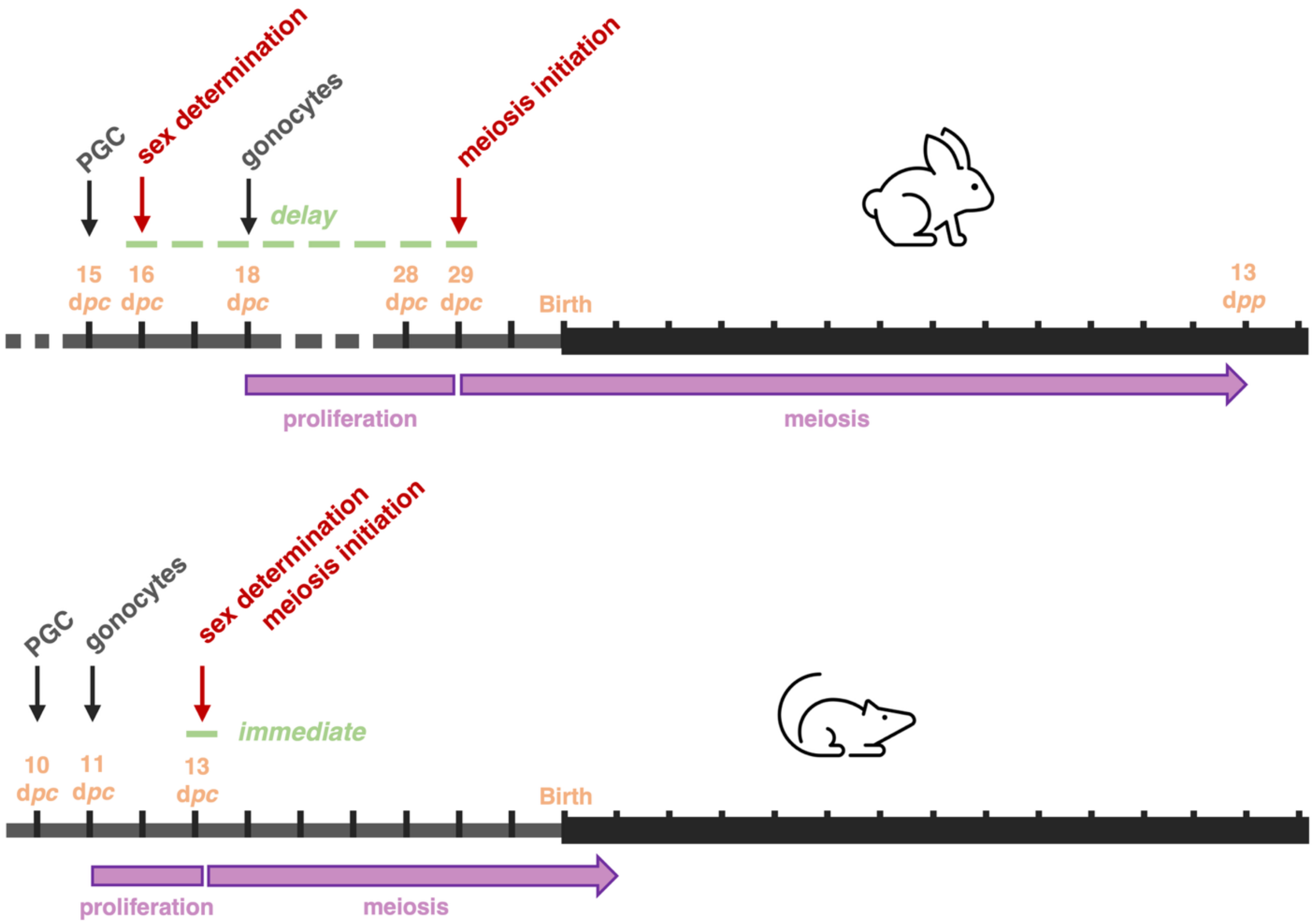

Fig. S1. Key steps of germ cell development during gonadal differentiation in rabbits compared to mice. PGC: primordial germ cells; d*pc*: days *post-coïtum*; d*pp*: days *post-partum*.

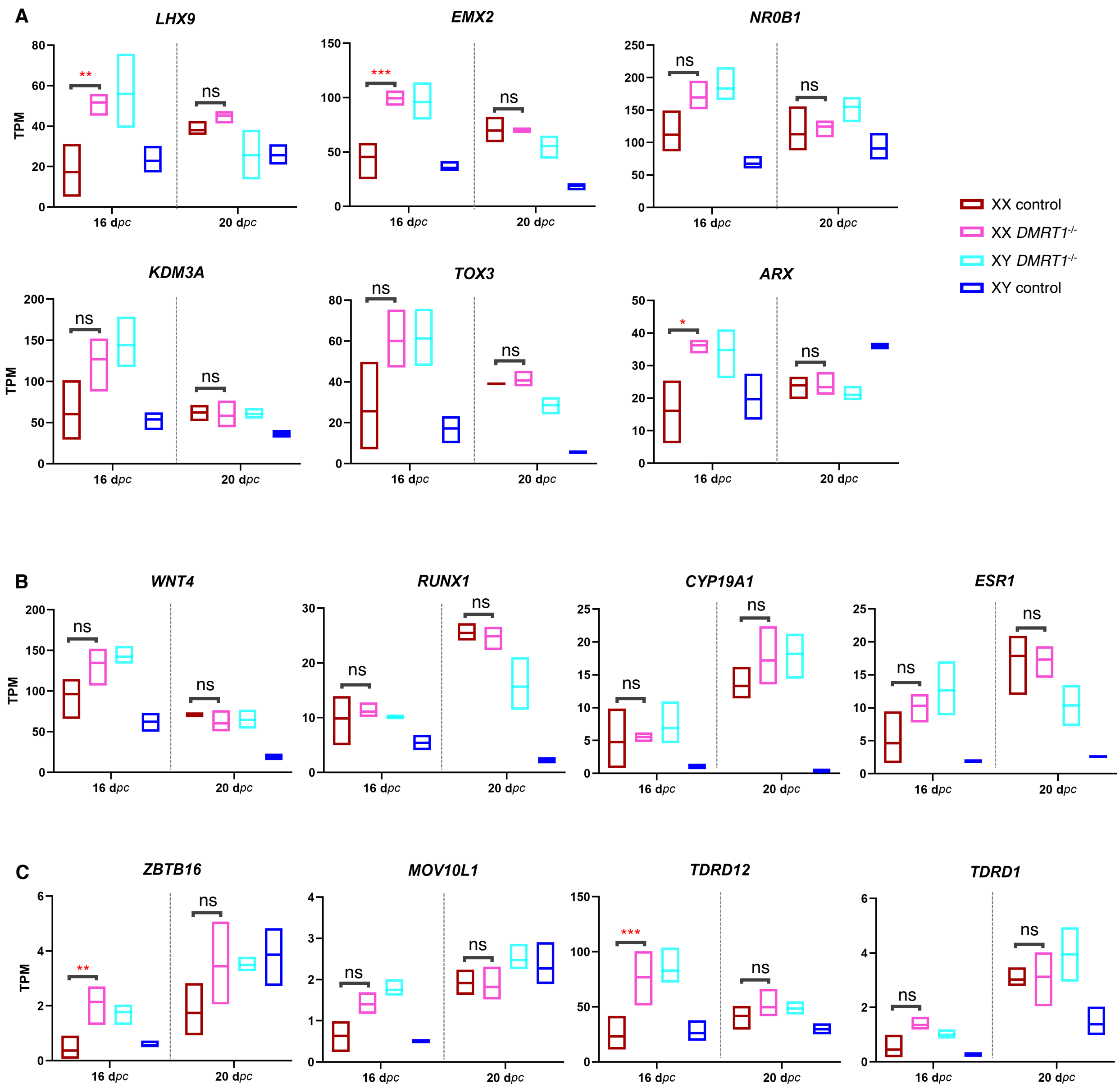

Fig. S2. Expression of key genes involved in gonadal development at 16 and 20 d*pc*. Expression data (in TPM: Transcripts Per kilobase Million) from RNA-sequencing data ($n = 3$) at 16 and 20 d*pc* on XX control, XX *DMRT1*$^{-/-}$, XY *DMRT1*$^{-/-}$ and XY control gonads for genes involved (**A**) in early gonadal differentiation (*LHX9*, *EMX2*, *NR0B1*, *KDM3A*, *TOX3*, and *ARX*), (**B**) in ovarian differentiation (*WNT4*, *RUNX1*, *CYP19A1*, and *ESR1*), or (**C**) in germ cells fate (*ZBTB16*, *MOV10L1*, *TDRD12*, and *TDRD1*). Statistical significance was determined using DESeq2 adjusted p-value: *p-value <0.05, **p-value <0.01, ***p-value <0.001, ns = non-significant.

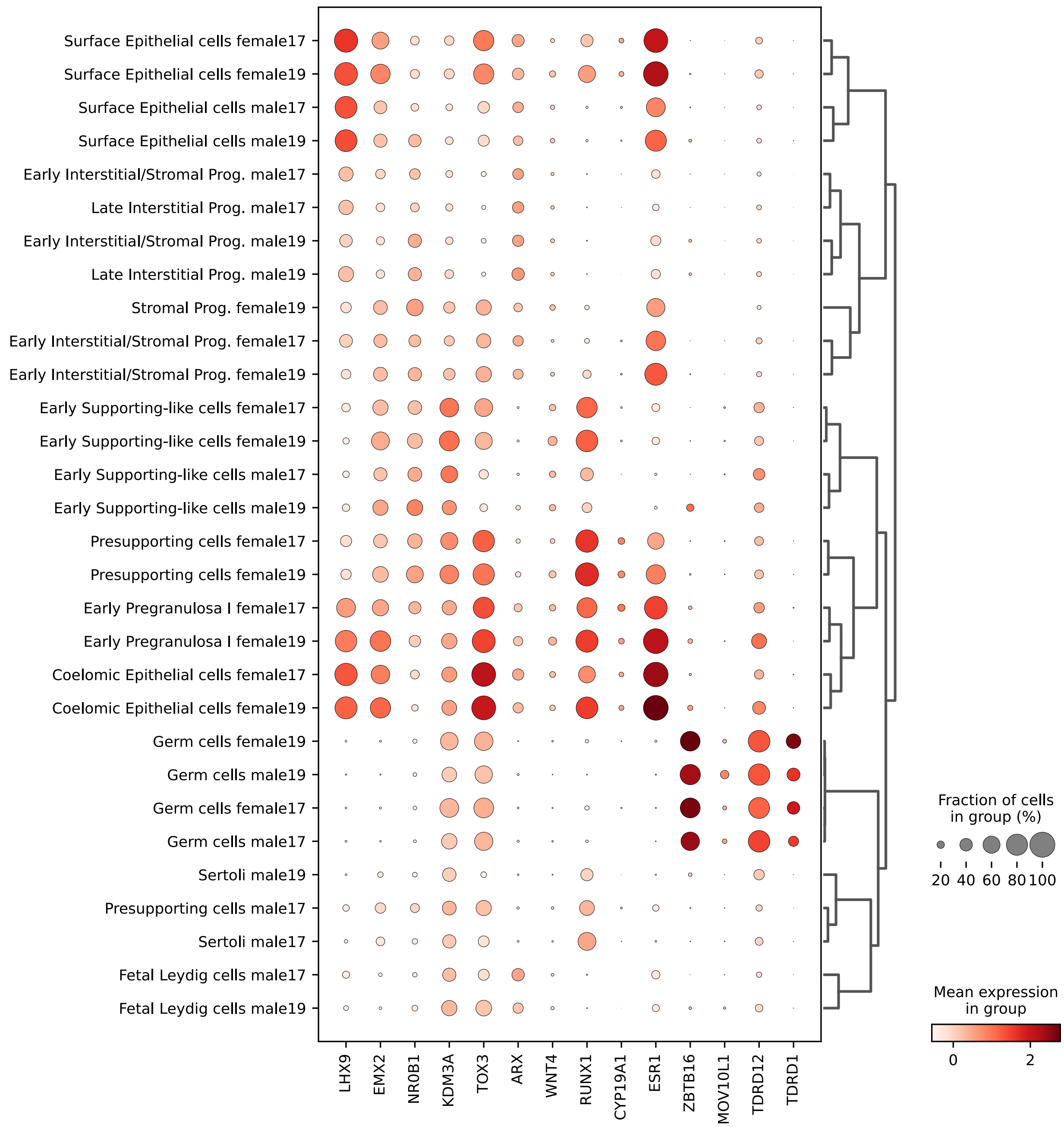

Fig. S3. Projection of selected genes onto single-cell RNA-seq data obtained on rabbit gonads at 17 and 19 d*pc*. Projection of genes from Fig. S2 to single-cell data from Barberá-Aura *et al.*, BioRxiv 2026.

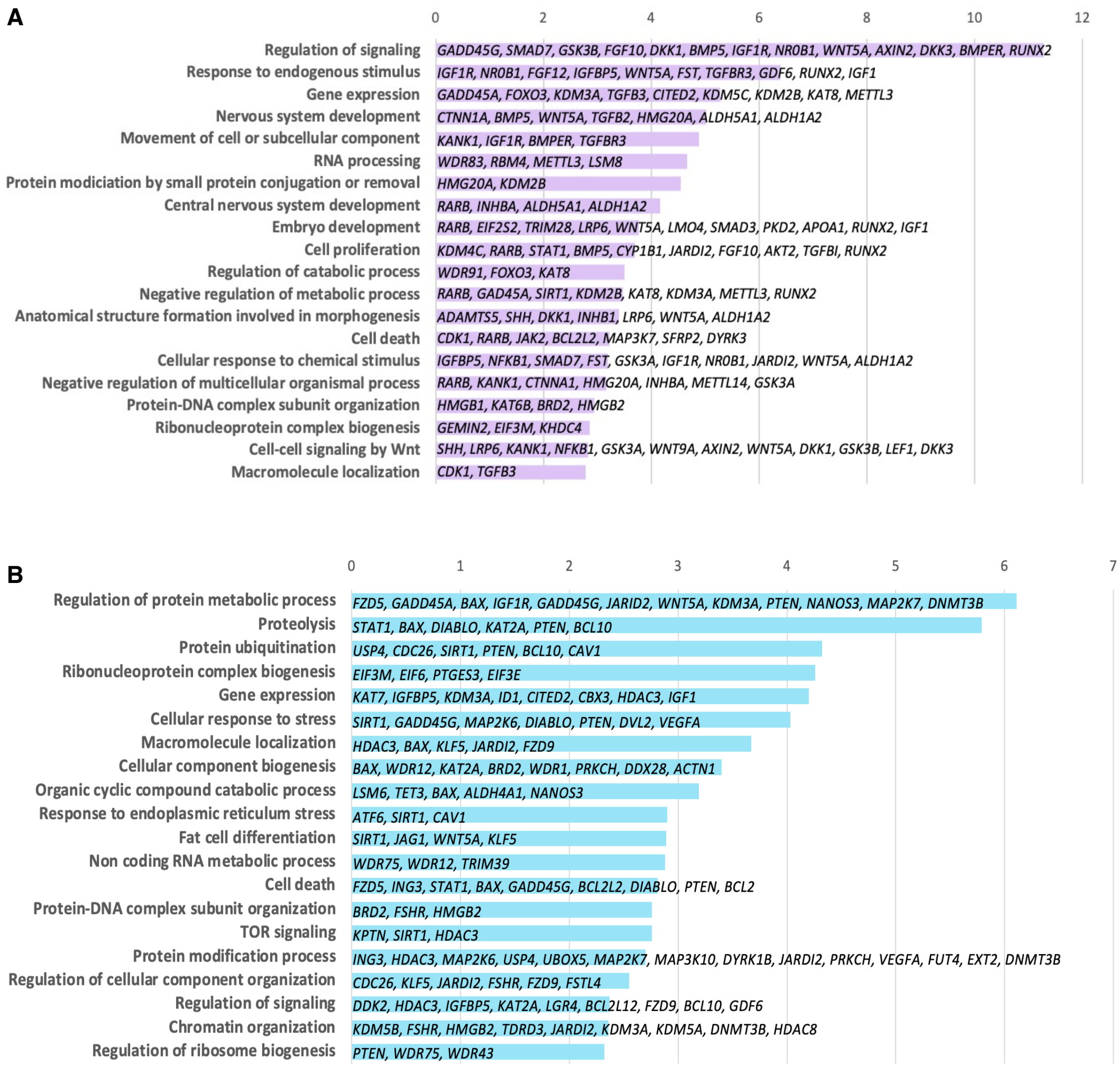

Fig. S4. Gene ontology of FOXL2 and DMRT1 targets in the rabbit gonads. The top twenty biological processes determined by DAVID analysis for the genes associated with (**A**) FOXL2 peaks in the ovary or (**B**) DMRT1 peaks in the testis. Terms are classed by their enrichment score, and the most relevant genes for each cluster are shown.

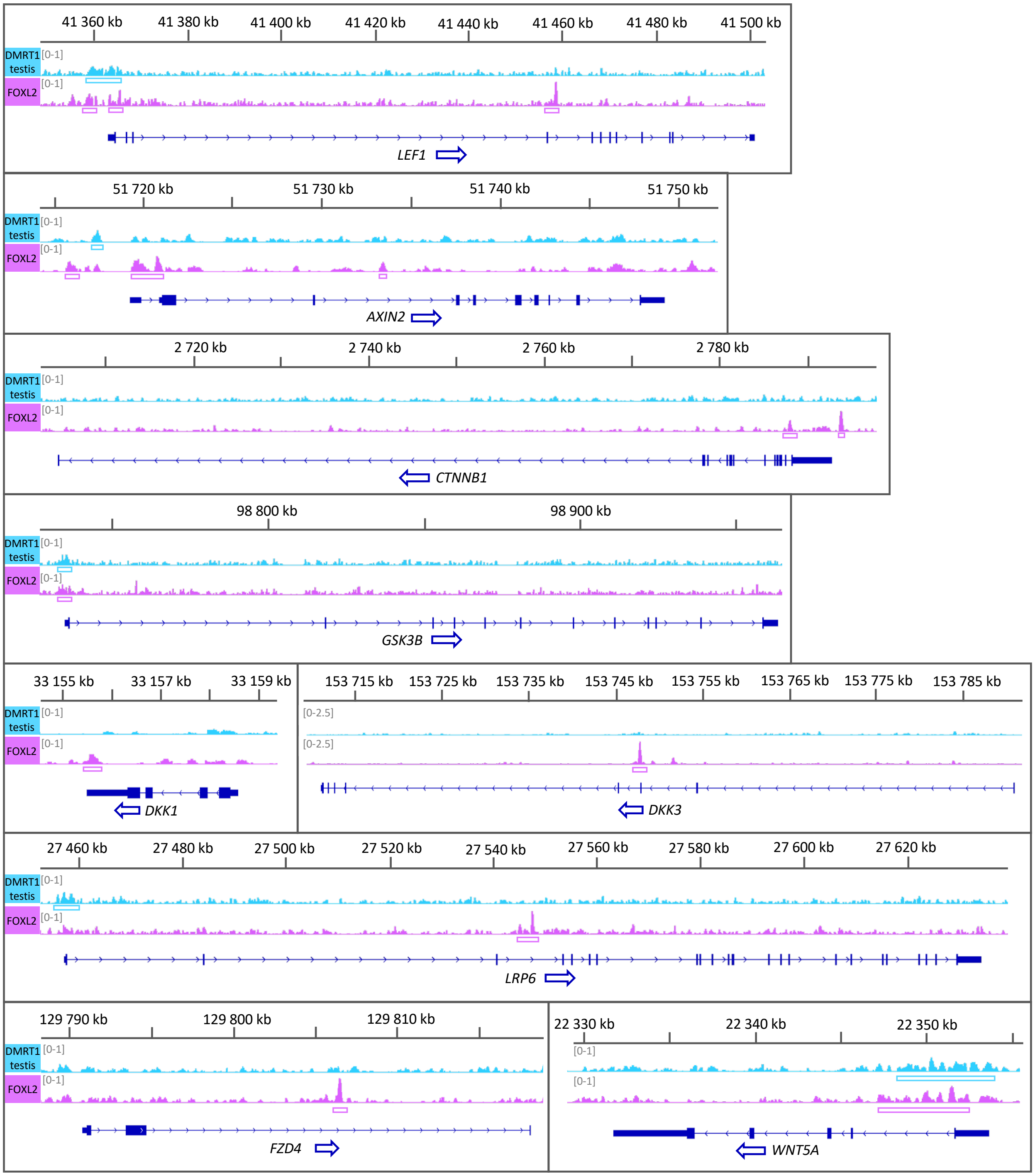

Fig. S5. Various components of the WNT/β-catenin pathway are targeted by DMRT1 and/or FOXL2 in rabbit gonads. Views of the IGV genome browser for components of the WNT/β-catenin pathway (*LEF1, AXIN2, CTNNB1, GSK3B, DKK1, DKK3, LRP6, FZD4,* and *WNT5A*) bound by DMRT1 in the rabbit testis (blue panel) and/or FOXL2 in the rabbit ovary (pink panel). For each, the input background was subtracted with IGV. The blue or pink rectangles underline relevant peaks.

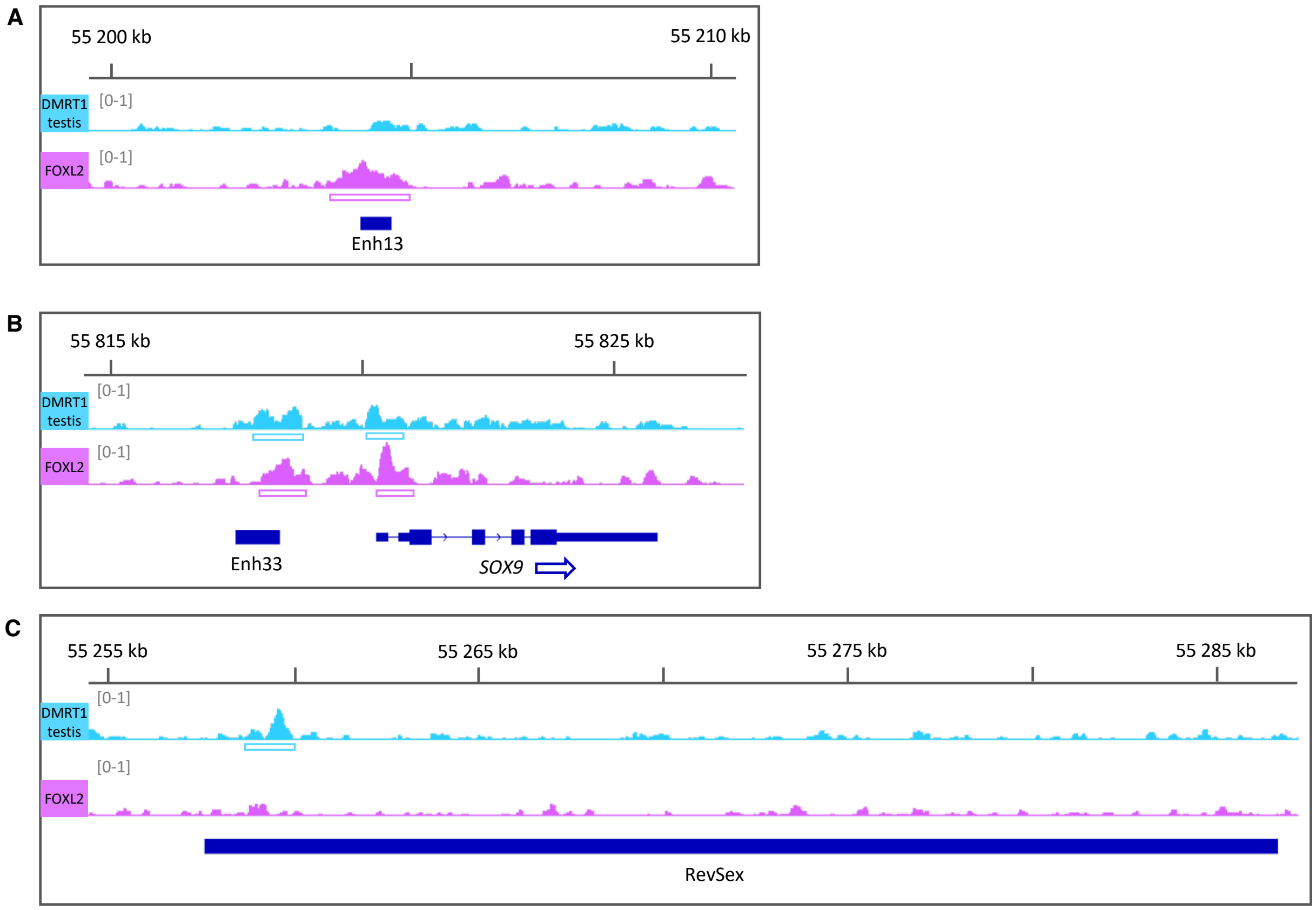

Fig. S6. Regulation of the *SOX9* expression in the rabbit gonads. Views of the IGV genome browser for the (**A**) Enhancer13 rabbit homologous region, (**B**) Enhancer33 rabbit homologous region and *SOX9* gene, or (**C**) the RevSex rabbit homologous region bound by DMRT1 in the rabbit testis (blue panel) and/or FOXL2 in the rabbit ovary (pink panel). For each, the input background was subtracted with IGV. The blue or pink rectangles underline relevant peaks.

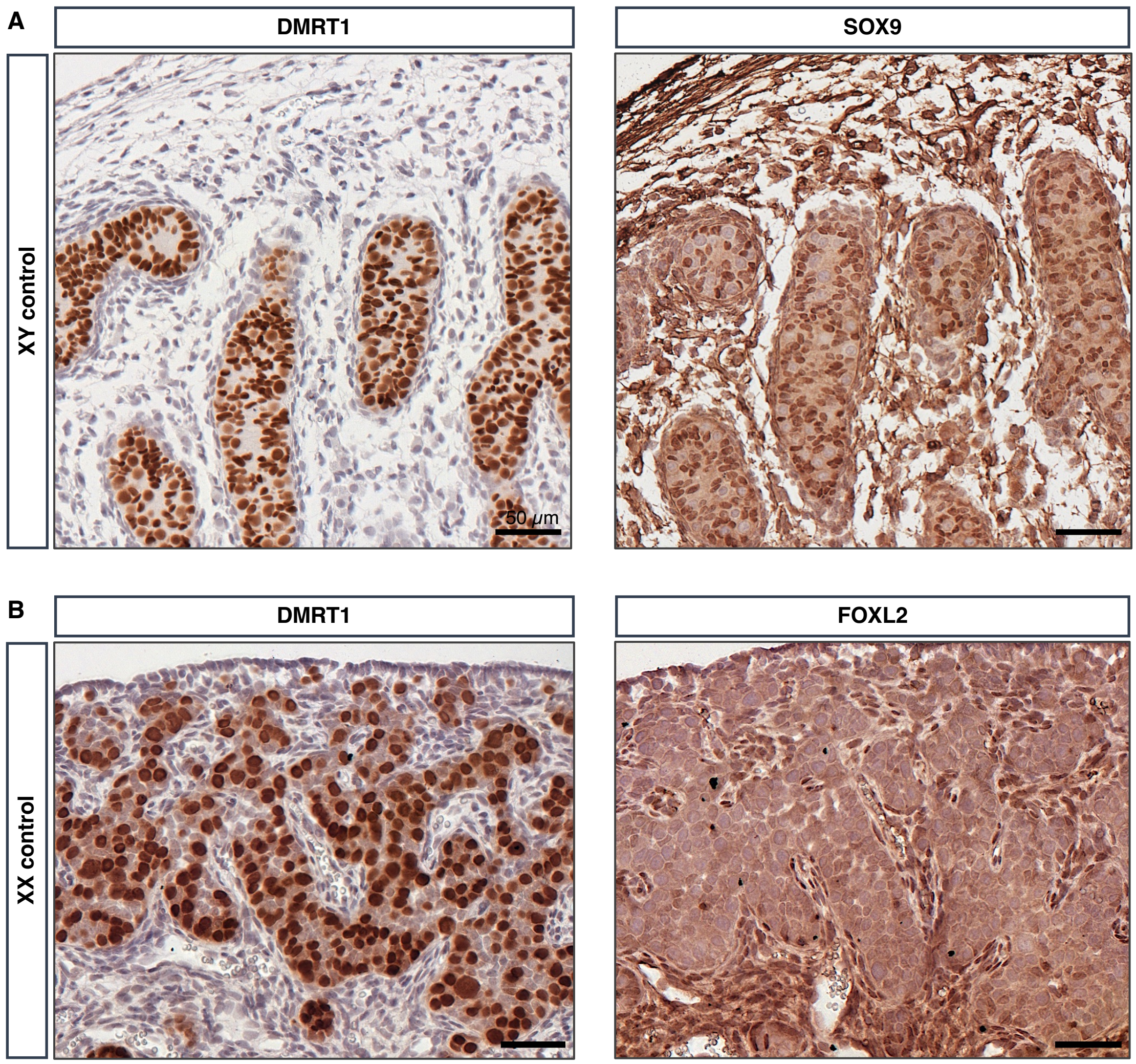

Fig. S7. Location of DMRT1, SOX9, and FOXL2 on 28 d*pc* rabbit gonads. (**A**) DMRT1 or SOX9 immunostaining on 28 d*pc* XY control gonads. (**B**) DMRT1 or FOXL2 immunostaining on 28 d*pc* XX control gonads. Scale bar = 50 μm.

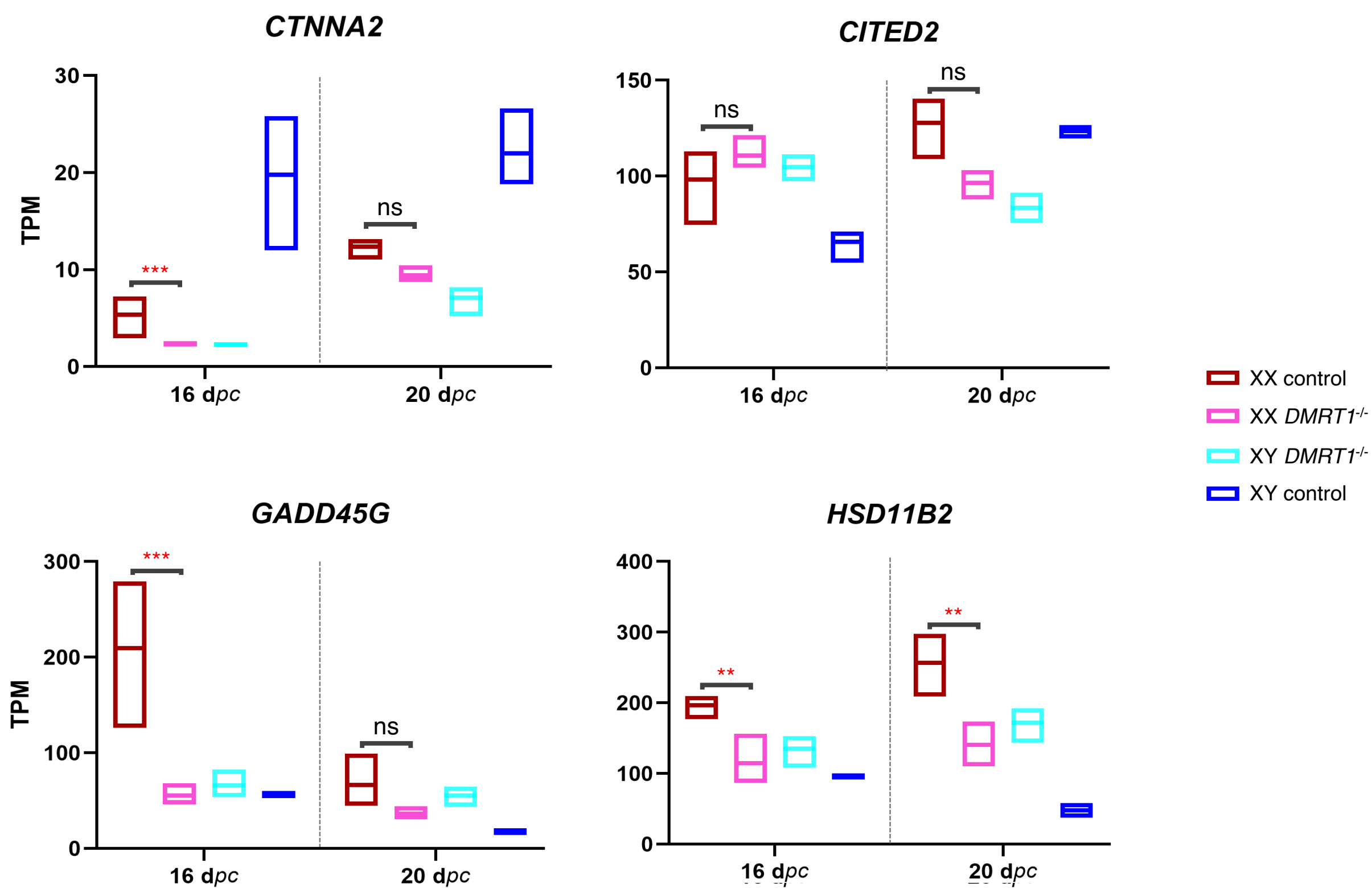

Fig. S8. Expression of *CTNNA2*, *CITED2*, *GADD45G,* and *HSD11B2* at 16 and 20 d*pc*. Expression data (in TPM) from RNA-sequencing data ($n = 3$) at 16 and 20 d*pc* on XX control, XX *DMRT1*$^{-/-}$, XY *DMRT1*$^{-/-}$, and XY control gonads. Statistical significance was determined using DESeq2 adjusted p-value: **p-value <0.01, ***p-value <0.001, ns = non-significant.

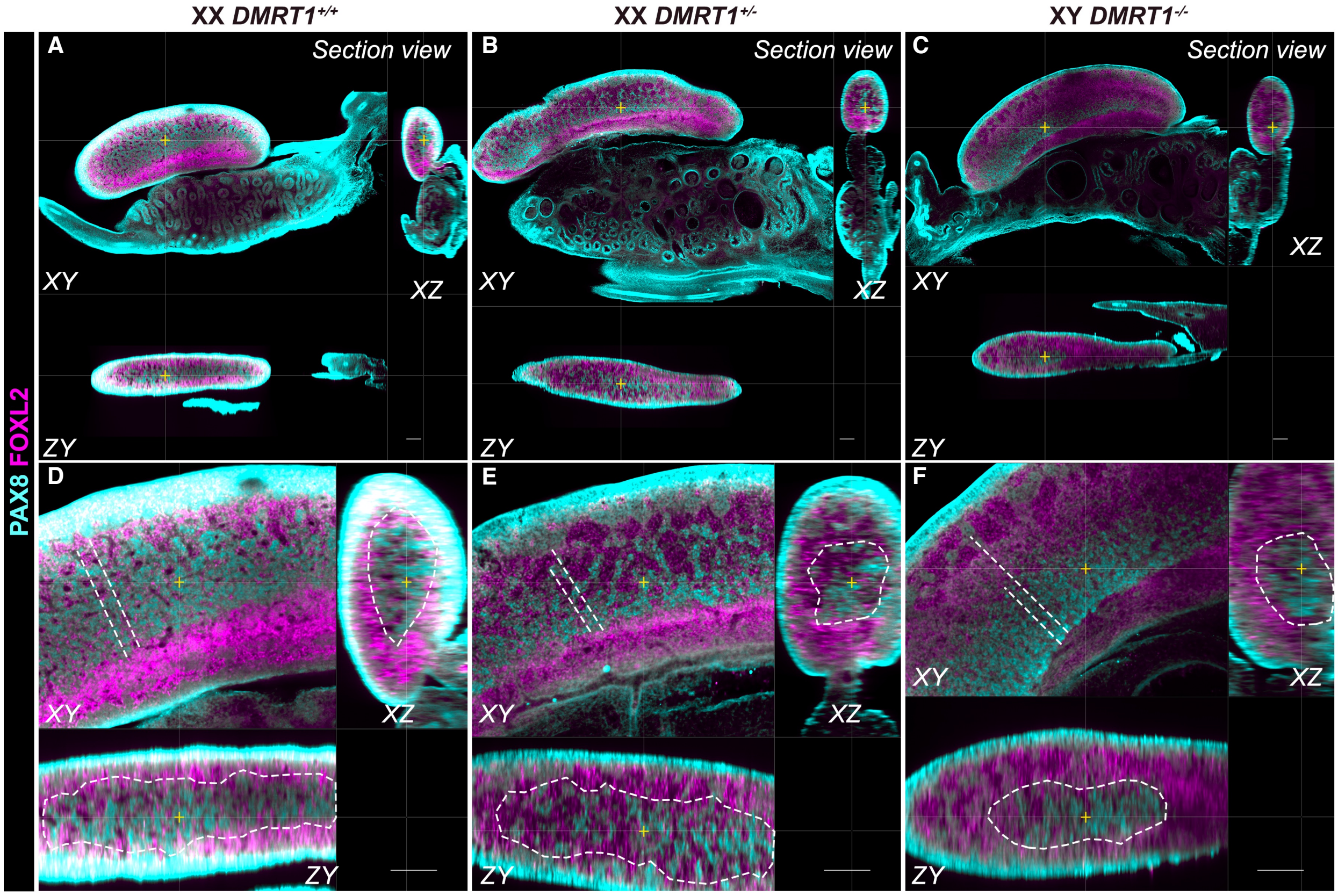

Fig. S9. The absence of DMRT1 affects colonization of PAX8+ cells across the ovarian medulla. Whole mount imaging of 22 d*pc* XX control (left), XX *DMRT1*$^{+/-}$ (middle), and XY *DMRT1*$^{-/-}$ (right) ovaries. **(A-C)**. Extended section view of X, Y, and Z axes across the volume of the whole ovary-mesonephros complexes labeled with PAX8 (cyan) and FOXL2 (magenta). **(D-F)** Zoomed-in views of the images in A-C show that the colonization of PAX8+ towards the ovarian cortex is hindered with the loss of DMRT1. White dotted straight lines show the extent of PAX8 expression (left line) vs. medullary width (right line), note the left line is shorter than the right line in XX *DMRT1*$^{+/-}$ and XY *DMRT1*$^{-/-}$ ovaries. The dotted circles illustrate outlines of the PAX8+ domain uses ZY and XZ projected views showing that the entire PAX8+ volume is less dense in XX *DMRT1*$^{+/-}$ and smaller in XY *DMRT1*$^{-/-}$ ovaries.

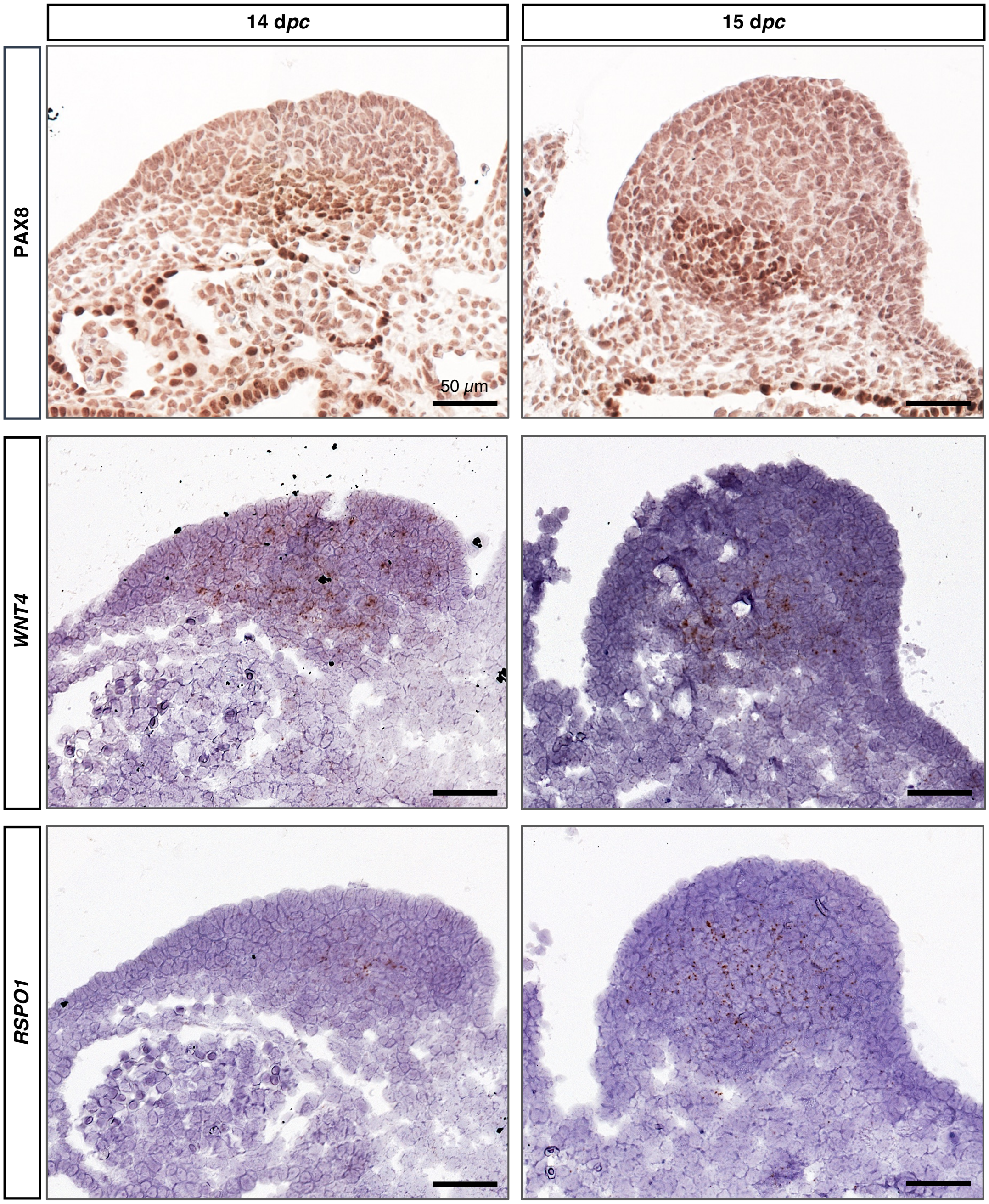

Fig. S10. Location of PAX8, *WNT4*, and *RSPO1*. PAX8 immunostaining and *in situ* hybridization (RNAscope technology) of *WNT4* and *RSPO1* on 14 and 15 d*pc* control ovaries. Scale bar = 50 µm.

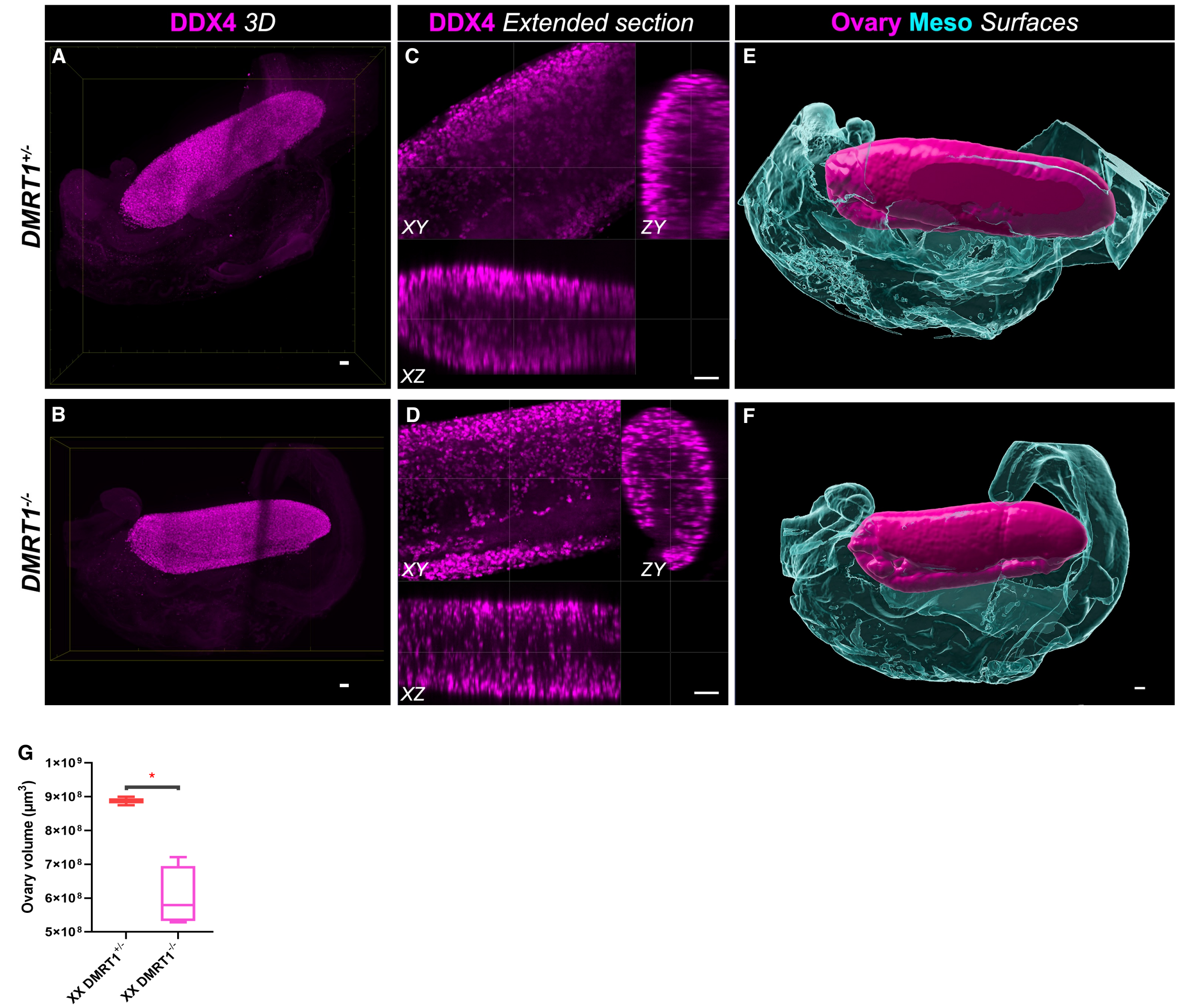

Fig. S11. Decrease of germ cell density in the ovarian cortex in the absence of DMRT1. Whole mount imaging of 4 d*pp* XX *DMRT1*$^{+/-}$ (top), and XX *DMRT1*$^{-/-}$ (bottom) ovaries. **(A-B)** 3D views of X, Y, and Z axes across the volume of the whole DDX4-labeled ovary (magenta). **(C-D)** Zoomed-in extended sections showing the density of DDX4+ germ cells is decreased with the loss of DMRT1. **(E-F)** 3D rendering of total ovary volume using the DDX4+ domain. **(G)** Comparison of the ovary volume (n = 2-4) between XX *DMRT1*$^{+/-}$ and XX *DMRT1*$^{-/-}$ gonads. Statistical analysis was performed using an unpaired t-test: * p-value <0.05.

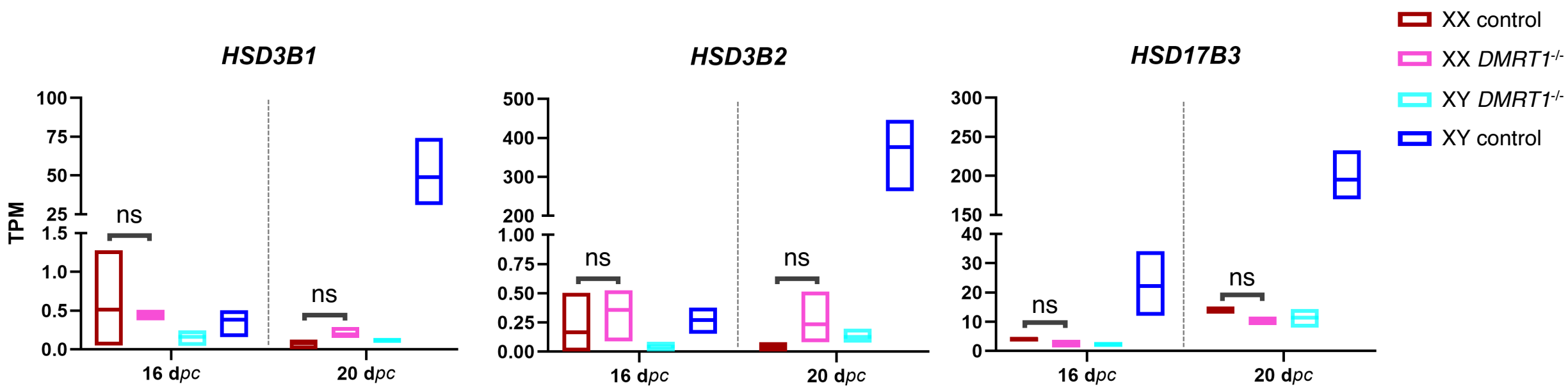

Fig. S12. Expression of steroidogenesis enzymes. Expression data (in TPM) from RNA-sequencing data (n = 3) at 16 and 20 d*pc* on XY control, XY *DMRT1*$^{-/-}$, XX *DMRT1*$^{-/-}$, and XX control gonads for genes encoding steroidogenesis enzymes (*HSD3B1, HSD3B2*, and *HSD17B3*). Statistical significance was determined using DESeq2 adjusted p-value: ns = non-significant.

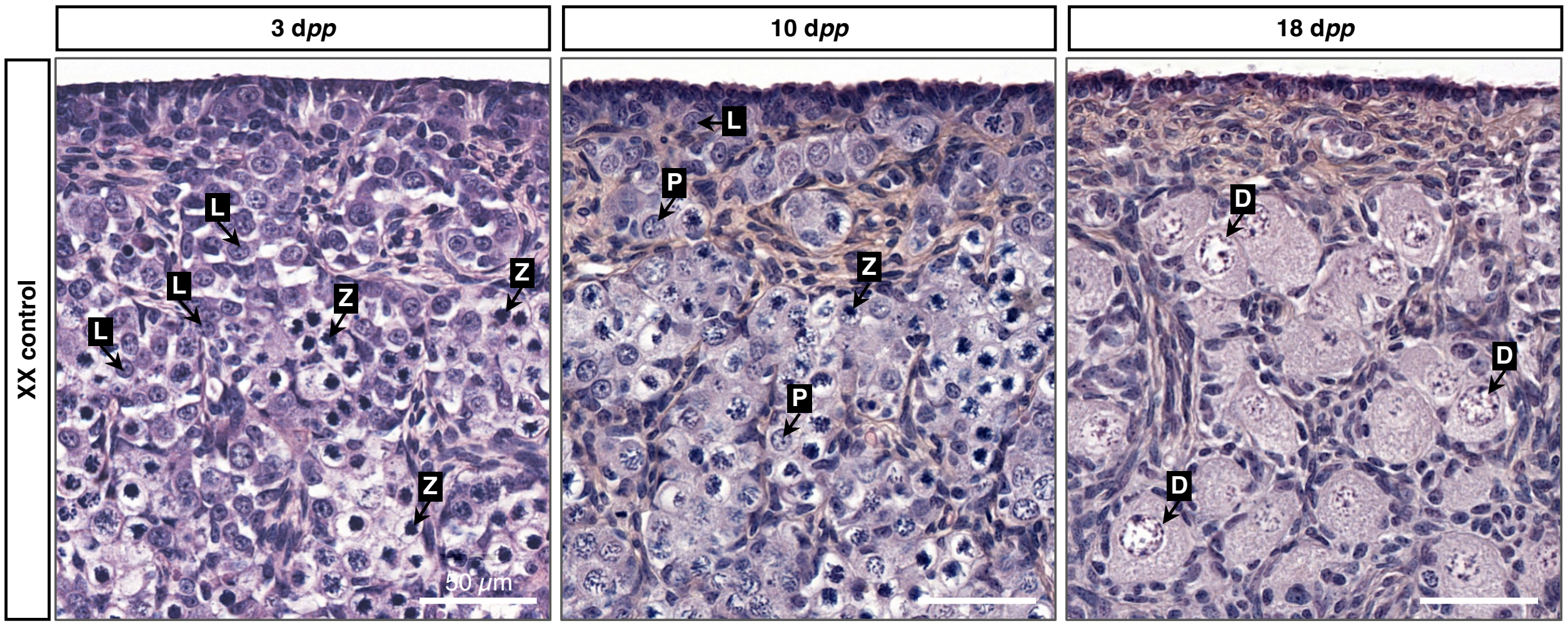

Fig. S13. Progression of the female germ cells into meiosis. Hematoxylin and eosin staining on XX control gonad sections from 3 to 18 d*pp*. L: leptotene stage; Z: zygotene stage; P: pachytene stage; D: diakinesis stage. Scale bar = 50 µm.

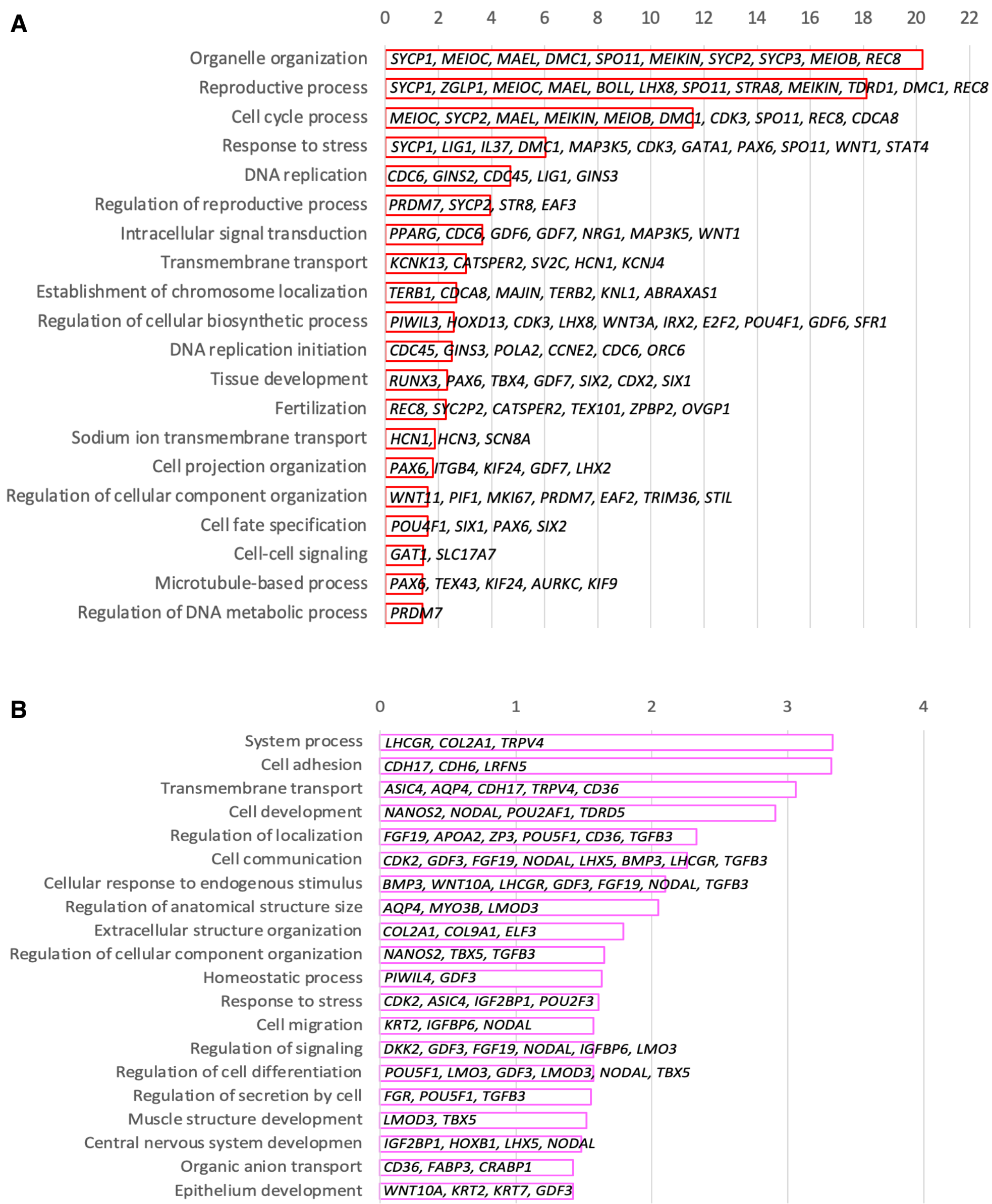

Fig. S14. Gene ontology of differentially expressed genes at 1 d*pp*. The top twenty biological processes determined by DAVID analysis for genes (**A**) down-regulated (adjusted p-value <0.05 and log2FC<-1) or (**B**) up-regulated in XX *DMRT1*$^{-/-}$ (adjusted p-value <0.05 and log2FC>1) gonads at 1 d*pp*. Terms are classed by their enrichment score, and the most relevant genes for each cluster are shown.

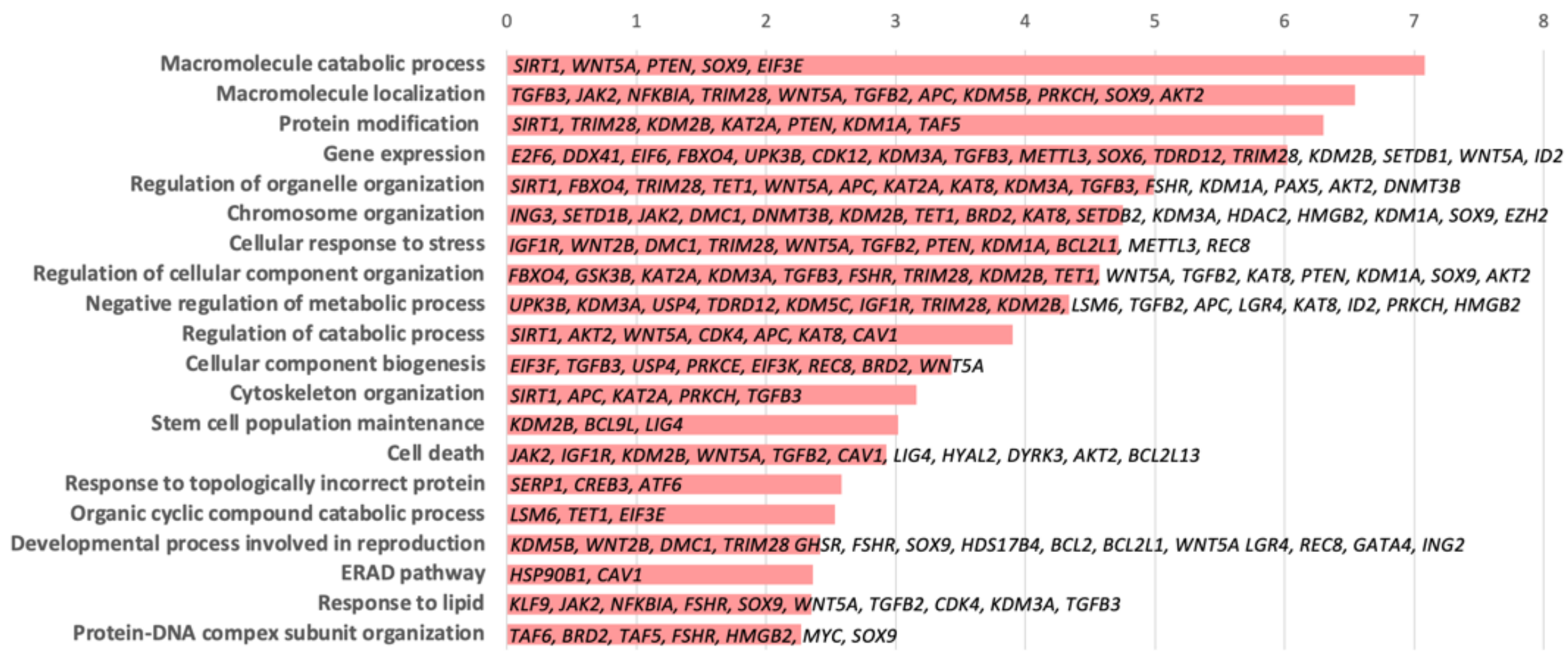

Fig. S15. Gene ontology of DMRT1 targets in the rabbit ovary. The top twenty biological processes determined by DAVID analysis for the genes associated with DMRT1 peaks. Terms are classed by their enrichment score, and the most relevant genes for each cluster are shown.

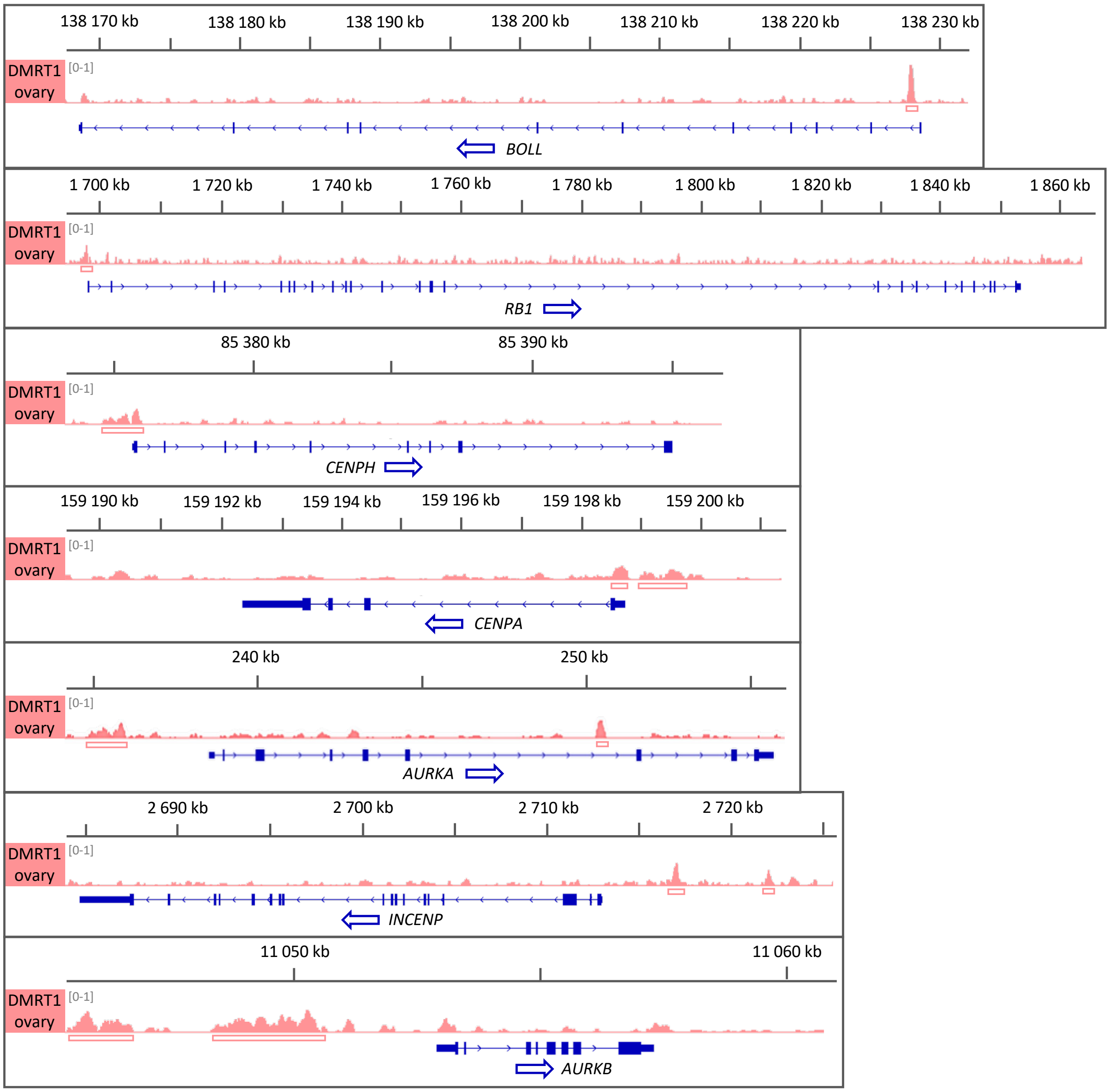

Fig. S16. Genes associated with the meiotic process are targeted by DMRT1 in the rabbit gonads. Views of the IGV genome browser for *BOLL, RB1, CENPH, CENPA, AURKA, INCENP,* and *AURKB* genes bound by DMRT1 in the rabbit ovary (salmon pink panel). The input background was subtracted with IGV. The rectangles underline relevant peaks.

Table S1. List of DEG in *DMRT1*$^{-/-}$ gonads (XY and XX) compared to control gonads (XY and XX) at 16 d*pc* (adjusted p-value < 0.05 and │log2FC│>1).

List of the differentially expressed genes (DEGs) between XY control vs XX control gonads (sheet 1), XX control vs XY *DMRT1*$^{-/-}$ (sheet 2), XX control vs XX *DMRT1*$^{-/-}$ gonads (sheet 3), and XY *DMRT1*$^{-/-}$ and XX *DMRT1*$^{-/-}$ (sheet 4). The gene clustering of the heatmap representation (Fig. 1C) is shown in sheet 5.

Table S2. List of the FOXL2 targets in the rabbit ovary and the gene ontology.

List of the FOXL2 annotated peaks in the rabbit ovary at 28 d*pc* (sheet 1). Functional annotation clustering from DAVID analysis (gene ontology term: biological process) on Ensembl IDs associated with FOXL2 targets (in intron) in the rabbit ovary (sheet 2). Functional annotation clustering from DAVID analysis (gene ontology term: biological process) on Ensembl IDs associated with FOXL2 targets (in exon, promoter-TSS, and TTS) in the rabbit ovary (sheet 3).

Table S3. List of the DMRT1 targets in the rabbit testis and the gene ontology.

List of the DMRT1 annotated peaks in the rabbit testis at 28 d*pc* (sheet 1). Functional annotation clustering from DAVID analysis (gene ontology term: biological process) on Ensembl IDs associated with DMRT1 targets in the rabbit testis (sheet 2). List of the common targets of DMRT1 in the testis and FOXL2 in the ovary at 28 d*pc* (sheet 3). Functional annotation clustering from DAVID analysis (gene ontology term: biological process) on Ensembl IDs associated with the common targets (sheet 4).

Table S4. Crossing of RNA-seq data with ChIP-seq data.

Comparison of the RNA-seq data considering the DEGs (adjusted p-value <0.05) obtained from the DESeq2 analysis with the ChIP-seq data for FOXL2 and DMRT1 in the testis. For the ChIP-seq data, we only considered the genes with peaks within the gene body. List of genes upregulated in XY control or XX control at 16 d*pc* that are common with FOXL2 peaks or DMRT1 peaks (sheet 1). List of genes upregulated in XX *DMRT1*$^{-/-}$ or XX control at 16 d*pc* that are common with FOXL2 peaks or DMRT1 peaks (sheet 2). List of genes upregulated in XY *DMRT1*$^{-/-}$ or XY control at 16 d*pc* that are common with FOXL2 peaks or DMRT1 peaks (sheet 3). List of genes upregulated in XY control or XX control at 20 d*pc* that are common with FOXL2 peaks or DMRT1 peaks (sheet 4). List of genes upregulated in XX *DMRT1*$^{-/-}$ or XX control at 20 d*pc* that are common with FOXL2 peaks or DMRT1 peaks (sheet 5). List of genes upregulated in XY *DMRT1*$^{-/-}$ or XY control at 20 d*pc* that are common with FOXL2 peaks or DMRT1 peaks (sheet 6).

Table S5. List of DEG in *DMRT1*$^{-/-}$ gonads (XY and XX) compared to control gonads (XY and XX) at 1 d*pp* (adjusted p-value < 0.05 and │log2FC│>1).

List of the differentially expressed genes (DEGs) between XX control and XX *DMRT1*$^{-/-}$ gonads (sheet 1). Functional annotation clustering from DAVID analysis (gene ontology term: biological process) on Ensembl IDs of genes with adjusted p-value < 0.05 and log2FC <-1 (sheet 2). Functional annotation clustering from DAVID analysis (gene ontology term: biological process) on Ensembl IDs of genes with adjusted p-value < 0.05 and log2FC >1 (sheet 3).

Table S6. List of the DMRT1 targets in the rabbit ovary and the gene ontology.

List of the DMRT1 annotated peaks in the rabbit ovary at 28 d*pc* (sheet 1). Functional annotation clustering from DAVID analysis (gene ontology term: biological process) on Ensembl IDs associated with DMRT1 targets in the rabbit ovary (sheet 2). List of genes that are DMRT1 targets in the rabbit ovary and down-regulated in XX *DMRT1*$^{-/-}$ gonads at 1 d*pp* (sheet 3). Functional annotation clustering from DAVID analysis (gene ontology term: biological process) on Ensembl IDs associated with DMRT1 targets in the rabbit ovary and genes down-regulated in XX *DMRT1*$^{-/-}$ gonads at 1 d*pp* (sheet 4).

Table S7. Synthesized probes used for *in situ* hybridization.

Gene name	RNAscope probe catalog number	Transcript accession number
WNT4	1059081	XM_008265713.2
RSPO1	488231	XM_002720657.2

Table S8. List of antibodies used for immunohistochemistry (IHC) or whole-mount immunofluorescence (WM IF).

	Antibody name	Reference	Dilution
Primary antibodies	Mouse monoclonal to DMRT1	sc-377167 (Santa Cruz)	1/500 (IHC)
	Rabbit polyclonal to PAX8	10336-1-AP (Proteintech)	1/6000 (IHC)
	Mouse monoclonal to PAX8	4H7B3 (Proteintech)	1/750 (WM IF)
	Goat polyclonal to hDDX4	AF2030 (R&D systems)	1/250 (WM IF)
	Goat polyclonal to FOXL2	ab5096 (abcam)	1/250 (WM IF)
Secondary antibodies	Biotinylated horse anti-rabbit	BA-1100 (Vector Laboratories)	1/200 (IHC)
	Biotinylated anti-mouse	Included in M.O.M. kit (BMK-2202, Vector Laboratories)	1/200 (IHC)
	Cy3 donkey anti-goat	705-165-147 (Jackson Immunolabs)	1/500 (WM IF)
	ATTO647 rabbit anti-mouse	610-456-C46 (Rockland)	1/750 (WM IF)

Table S9. Primers used for RT-qPCR analyses.

Gene	Forward (5'-3')	Reverse (3'-5')
RSPO1	GCCCGCCTGGATACTTCGA	GGTGCAGAAGTTGTGGCTGAA
DKK1	CAATCTGTCTGGCTTGCAG	GGCATGCATATTCCGTTTTT
DDX4	CAACTCGAGAGCTGATCAACCA	CCCAACTGGGTTCCTCCATA
POU5F1	CCGACAACAATGAGAACCTTCAG	TTGCCTCTCACTCGGTTCTCAA
DAZL	CAGTTCACGAAGCTGCTCCAT	TCAGCCTGTTCTCAGGATTAAACA
STRA8	GACAACAATGAGGCTCCAAATG	TGATCTGCACGTAGAGCTGAAAC
HPRT	AGTTTGTTGTAGGATATGCCCTTG	TAGGCTTTGTATTTTGCTTTTCCA
H2AFX	ACCTGACGGCCGAGATCCT	CGCCCAGCAGCTTGTTGAG
YWHAZ	GGGTCTGGCCCTTAACTTCTCT	AGCAATGGCTTCATCAAAAGC
SF1 (splicing Factor 1)	GCTTCCGACTGCAAATTCCA	TCACCCAGTTCAGCCATGAG

Movie S1. Whole-mount immunofluorescence for PAX8 and FOXL2 on XX *DMRT1*$^{+/+}$ gonad at 22 d*pc*.

Movie S2. Whole-mount immunofluorescence for PAX8 and FOXL2 on XX *DMRT1*$^{+/-}$ gonad at 22 d*pc*.

Movie S3. Whole-mount immunofluorescence for PAX8 and FOXL2 on XY *DMRT1*$^{-/-}$ gonad at 22 d*pc*.

Movie S4. Whole-mount immunofluorescence for PAX8 and DDX4 on XX *DMRT1*$^{+/-}$ gonad at 4 d*pp*.

Movie S5. Whole-mount immunofluorescence for PAX8 and DXX4 on XX *DMRT1*$^{-/-}$ gonad at 4 d*pp*.
